# INTREPID II: protocol for a multistudy programme of research on untreated psychosis in India, Nigeria and Trinidad

**DOI:** 10.1136/bmjopen-2020-039004

**Published:** 2020-06-21

**Authors:** Tessa Roberts, Oye Gureje, Rangaswamy Thara, Gerard Hutchinson, Alex Cohen, Helen Anne Weiss, Sujit John, Joni Lee Pow, Casswina Donald, Bola Olley, Georgina Miguel Esponda, Robin M Murray, Craig Morgan

**Affiliations:** 1 Health Service & Population Research department, Institute of Psychiatry Psychology and Neuroscience, London, UK; 2 ESRC Centre for Society and Mental Health, King's College London, London, UK; 3 WHO Collaborating Centre for Research and Training in Mental Health, Neuroscience and Substance Abuse, Department of Psychiatry, University of Ibadan, Ibadan, Oyo, Nigeria; 4 Schizophrenia Research Foundation, Chennai, India; 5 Department of Psychiatry, The University of the West Indies at Saint Augustine Faculty of Medical Sciences, Saint Augustine, Tunapuna–Piarco, Trinidad and Tobago; 6 Department of Epidemiology, Harvard University T H Chan School of Public Health, Boston, Massachusetts, USA; 7 Epidemiology and Population Health, London School of Hygiene and Tropical Medicine, London, UK; 8 London School of Hygiene and Tropical Medicine, London, UK; 9 Department of Psychiatry, University of Ibadan College of Medicine, Ibadan, Oyo, Nigeria; 10 Department of Psychosis Studies, Institute of Psychiatry Psychology and Neuroscience, London, UK

**Keywords:** schizophrenia & psychotic disorders, epidemiology, mental health

## Abstract

**Introduction:**

There are few robust and directly comparable studies of the epidemiology of psychotic disorders in the Global South. INTREPID II is designed to investigate variations in untreated psychotic disorders in the Global South in (1) incidence and presentation (2) 2-year course and outcome, (3) help-seeking and impact, and (4) physical health.

**Methods:**

INTREPID II is a programme of research incorporating incidence, case–control and cohort studies of psychoses in contiguous urban and rural areas in India, Nigeria and Trinidad. In each country, the target samples are 240 untreated cases with a psychotic disorder, 240 age-matched, sex-matched and neighbourhood-matched controls, and 240 relatives or caregivers. Participants will be followed, in the first instance, for 2 years. In each setting, we have developed and are employing comprehensive case-finding methods to ensure cohorts are representative of the target populations. Using methods developed during pilot work, extensive data are being collected at baseline and 2-year follow-up across several domains: clinical, social, help-seeking and impact, and biological.

**Ethics and dissemination:**

Informed consent is sought, and participants are free to withdraw from the study at any time. Participants are referred to mental health services if not already in contact with these and emergency treatment arranged where necessary. All data collected are confidential, except when a participant presents a serious risk to either themselves or others. This programme has been approved by ethical review boards at all participating centres. Findings will be disseminated through international conferences, publications in international journals, and through local events for key stakeholders.

**Strengths and limitations of this study**

- Comprehensive case-finding methods, building on extensive pilot work, to generate as complete a sample as possible and reduce selection bias.
- Inclusion of population-based, matched controls.
- Direct comparability of methods across settings.
- Potential trade-offs between cross-setting comparability and local validity.
- Use of retrospective self-reports for several factors, which are potentially subject to recall bias and which create challenges in establishing the direction of associations.

## Introduction

Psychotic disorders, such as schizophrenia, affect more than 23 million people worldwide, contribute substantially to the global burden of disease and are associated with high rates of disability and mortality.^1–3^ However, there are striking global inequities in our knowledge of psychoses. Over 85% of the world’s population lives in Asia, Africa, Latin America and the Caribbean (referred to here as the Global South; the term Global South refers to countries in Asia, Africa, Latin America and the Caribbean and does not necessarily refer to the geographical south, see eg, http://www.fc-ssc.org/en/partnership_program/south_south_countries), but only a small fraction of research on psychotic disorders is done in these settings.^4 5^ This has two implications. First, our knowledge of psychotic disorders, especially of the basic epidemiology, of associated risk factors, and of course and outcome, is incomplete and may be distorted. We do not know whether psychoses manifest, occur and develop in the same ways around the world. Second, we do not have robust and replicated findings on which to base the development of accessible, humane and effective services and public health initiatives in low resource settings. Conducting studies in a range of countries and contexts is essential to improve our understanding of the nature of psychotic disorders globally and to provide a much-needed evidence base to inform the development and implementation of effective interventions and services in diverse settings.

We established the International Programme of Research on Psychotic Disorders (INTREPID) II—the first multicountry study in four decades in the Global South—to extend our knowledge of psychotic disorders in diverse settings. This builds on extensive feasibility and pilot work^5–7^ (INTREPID I; see Supplementary Materials, online supplementary appendix 1).

10.1136/bmjopen-2020-039004.supp1 Supplementary data

## Aim, objectives and rationale

Our aim is to investigate variability in incidence, presentation, outcome and impact of untreated psychotic disorders in three diverse countries of the Global South—India, Nigeria and Trinidad—through four interconnected studies.

### Study 1: incidence, presentation and risk

Objective: To investigate the incidence and presentation of untreated psychotic disorders in each setting and associated risk factors.

Psychotic disorders are highly heterogenous in incidence, presentation and course and outcome. For example, the incidence of schizophrenia and other psychoses varies markedly across populations and social groups.^8 9^ Rates are higher among men,^9^ in urban areas,^10^ and in many—but not all—migrant and minority ethnic populations.^11^ However, little is known about the incidence of psychoses in the Global South, beyond a small number of studies,^5 12^ and we cannot assume that findings from the Global North generalise to other settings. There is tentative evidence, for example, that findings from the Global North, such as the association with urbanicity, may not apply universally.^13–15^ Further, the phenomenology (ie, symptom profile) of psychotic disorders is highly varied. Individuals experience a range of symptoms, in various combinations, spanning multiple dimensions, including symptoms of reality distortion (ie, delusions, hallucinations), thought disturbance, mania, depression and poverty of affect, speech and volition. There is some evidence that symptom profiles vary across social and cultural contexts. For example, the Determinants of Outcome of Severe Mental Disorders (DOSMeD) study, a 2-year cohort study conducted in 10 countries by the WHO, found that non-affective acute remitting psychoses (ie, presentations characterised by rapid onset, symptoms of reality distortion and quick remission) were around 10 times more common in settings in the Global South compared with the Global North,^16^ but these findings have not been replicated.

There is robust evidence from the Global North implicating an array of factors that likely combine in complex ways to increase risk. These include genetic,^17 18^ neurodevelopmental markers (eg, birth complications, poor premorbid function),^19 20^ exposure to trauma and other social disadvantages,^21 22^ migration and minority ethnic status^11 23^ and substance use.^24 25^ Further, there is growing evidence that specific risk factors are associated with particular symptoms.^18 26–28^ For example, there is evidence of an association between social risk factors and specific symptoms of reality distortion,^29–36^ that is, more delusions and hallucinations. It may be, then, that variations in incidence and presentation between settings reflect different population distributions in relevant risks. However, little research has explored these associations in the Global South.

Studying variations in incidence and presentation and associated risks in diverse populations may provide important insights into the aetiology of psychoses and provide a basis for developing public health strategies to reduce the burden of psychotic disorders. In this study, we will test several primary hypotheses on whether variations and associations observed in the Global North hold in more diverse settings.

### Study 2: course and outcome

Objective: To investigate 2-year course and outcome of psychotic disorders and associated factors.

The long-term course and outcome of psychoses following a first episode is highly variable. Evidence from the Global North suggests that, over a period of 5–10 years, around half of those with a psychotic disorder recover symptomatically (ie, are symptom free for a period of 2 or more years),^37–40^ but the proportion who achieve both symptom and social recovery is much lower (8%–20%),^41^ with high levels of enduring unemployment and social isolation.^42–47^ Several factors are associated with poor symptom and social outcomes, including premorbid difficulties, baseline symptom type (ie, negative symptoms) and severity, cognition, long duration of untreated psychosis and persistent substance use.^48–50^ As with incidence and presentation, it seems that course and outcome vary by context. The DOSMeD study^51^ and the International Study of Schizophrenia (ISoS)^12^ reported better symptom and social outcomes for psychotic disorders in developing (ie, Global South) versus developed (ie, Global North) countries, which has often been attributed to greater family support and community cohesion in more traditional societies. There are, however, several well-documented methodological limitations to the DOSMeD and ISoS, not least that the number of countries included from the Global South is small (n=3). Subsequent research appears to show greater variation between and within countries in the Global South.^52^

In this study, we will describe and compare course and outcome at 2 years within and between settings, and then test several primary hypotheses on the nature and origins of any observed variations.

### Study 3: help-seeking and impact

Objective: To investigate: (1) help-seeking and (2) the impact of psychotic disorders on individuals and families, using a combination of quantitative and qualitative approaches.

Many people with psychotic disorders in the Global South receive no formal (biomedical) treatment or begin treatment well after the critical window when early intervention is most effective.^6 53^ Formal care in many countries often falls below minimum quality standards,^54^ and much of the burden of care falls on families. The use of traditional and religious healing for mental health problems is widespread in both Africa and Asia, even among those who also consult mental health services.^55 56^ Such services also exist in the Caribbean, but are more disparate, less specialised and typically used in addition—rather than as an alternative— to formal health services.^5 6^ Practitioners of traditional medicine and faith healing fill a major gap in countries where formal care is scarce,^57^ but the nature and quality of the care they provide is highly variable.^58^ Human rights abuses have been widely documented in both traditional healing sites and formal mental health services around the world.^59^ In part because of this, family members provide a large proportion of care for people with long-standing problems—including severe mental disorders—in the Global South.^60^ Caring for a relative with a psychotic disorder can have a major physical, emotional and economic impact on families, particularly in households with limited resources.^61–63^ There is also evidence of high levels of stigma in many countries of the world, including India,^64^ Nigeria^65^ and Trinidad.^66^

To plan appropriate services and understand differences in outcomes, further in-depth evidence, both quantitative and qualitative, is needed about how individuals and families respond to psychotic disorders and their needs and experiences, including the treatment they receive, within local contexts. In this study, we will first describe and compare, between and within settings (eg, by gender, by age and so on), the types and extent of contacts with formal services and other providers and the impact (ie, on quality of life) and burden of psychoses for individuals and families. We will then test, using quantitative data, several related primary hypotheses and address, using in-depth qualitative data, questions concerning how individuals and families make sense of and respond to psychoses and the impacts on individuals and families.

### Study 4: physical health

Objective: To investigate the types and prevalence of physical health problems and related biological markers.

In the Global North, those with a psychotic disorder have higher rates of physical health problems and higher rates of all-cause mortality,^67^ particularly cardiovascular disease and metabolic syndrome,^68^ which may result from both antipsychotic medication use and lifestyle factors.^69–71^ Comorbidity of physical and mental health problems is likely to impact negatively on quality of life and recovery.^72^ Our knowledge of the physical health of people with psychoses in the Global South is much more limited,^73^ but suggests that there is also a mortality gap compared with the general population and this may be related to similar health problems as in the Global North.^74 75^ For example, evidence from India suggests that metabolic syndrome is common^76^ and there are rising rates of diabetes and cardiovascular disease in India,^77^ Nigeria^78^ and Trinidad.^79^ It may also be, however, that the types of physical health problems (eg, malnutrition; infectious diseases; injury due to accident, violence) in developing countries differ from those common in developed countries.

In this study, we will describe and compare, between and within settings, markers and measures of physical health problems between cases and age-matched and sex-matched controls, and test hypotheses concerning the nature and origins of variations in physical health within and between settings.

## Feasibility and pilot work

See online supplementary appendix 1 in our Supplementary Materials for a description of our feasibility and pilot work.

## Settings

INTREPID II is a collaboration between the Schizophrenia Research Foundation (SCARF; Chennai), the University of Ibadan (Nigeria), the University of the West Indies at St Augustine (Trinidad), the London School of Hygiene and Tropical Medicine (UK) and the Institute of Psychiatry, Psychology & Neuroscience, King's College London (UK).

The study settings, in India, Nigeria and Trinidad, were selected to maximise potential comparisons between sites and with existing datasets. They represent three economically, socially and culturally diverse areas, on three continents, each undergoing rapid economic and social transformations.

In each setting, our catchment areas comprise urban and rural areas with total populations of around 500 000 adults aged 18–64 years. In Nigeria, the catchment area comprises three contiguous Local Government Areas in and around the city of Ibadan in Oyo State: Ibadan North East, Ibadan South East and Ona-Ara (total adult population ~584 000, population density 914–18 356 per km^2^). In Trinidad, the catchment area comprises the municipalities of Arima, Tunapuna–Piarco, Chaguanas, Port of Spain, San Juan/Laventille, Diego Martin and Sangre Grande (total adult population ~487 000, population density 82–3090 per km^2^). In India the catchment area consists of three contiguous taluks, Chengalpattu, Uthiramerur and Maduranthakam, located south of Chennai, in the district of Kancheepuram in the state of Tamil Nadu (total adult population ~600 000, population density 361–737 per km^2^).

## Methods

### Overview

**Figure 1:**
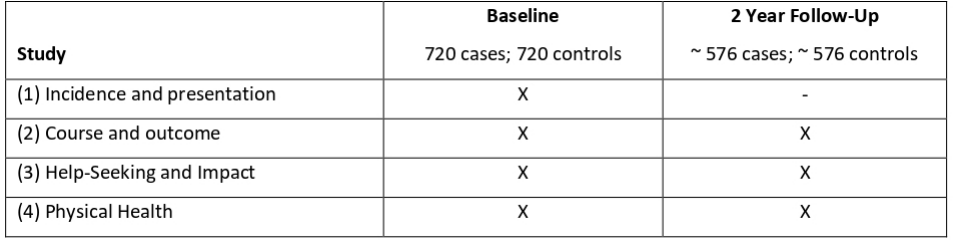
Structure of INTREPID II.

**Figure 2:**
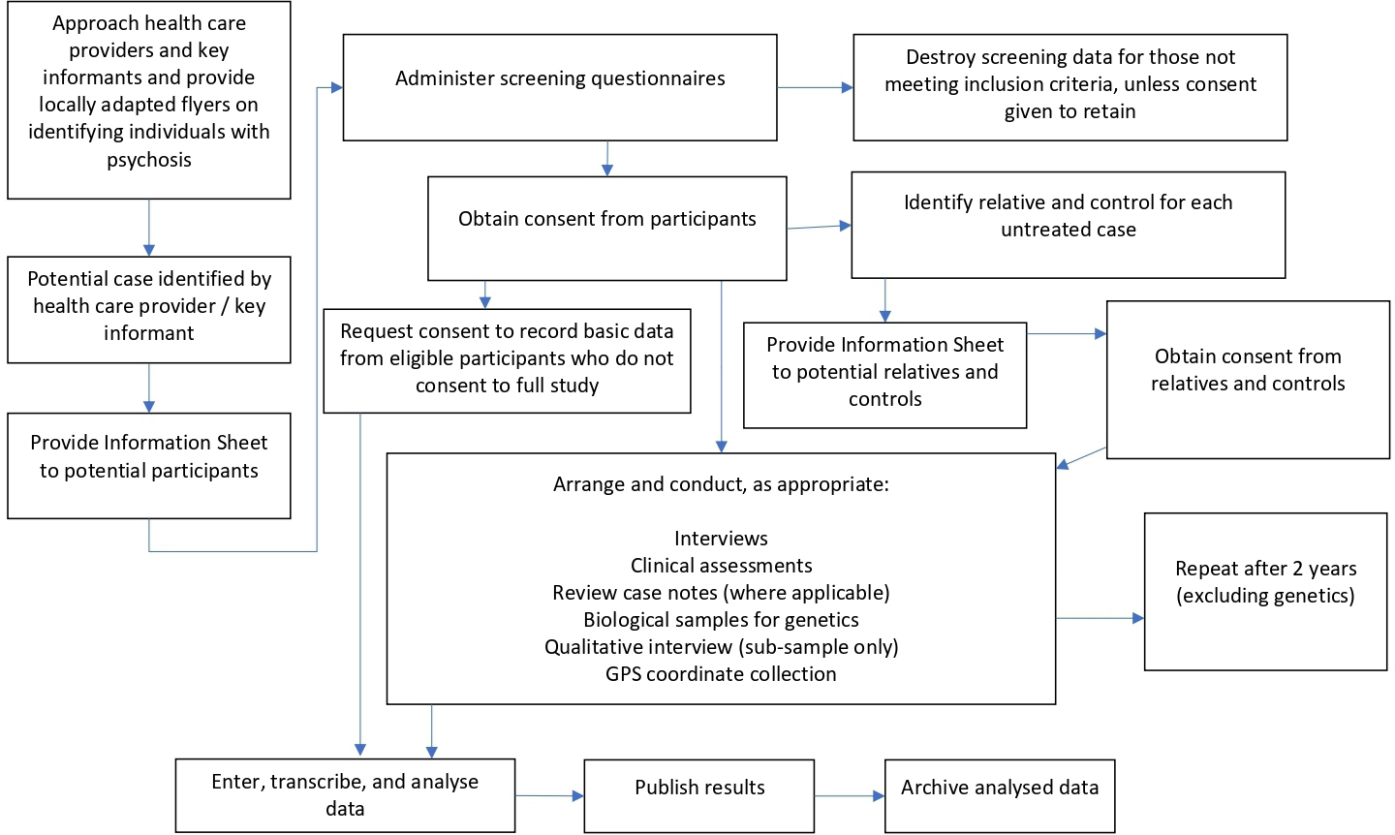
Summary of methodology.

INTREPID II comprises four interconnected studies (figure 1; see Strobe Statement, online supplementary materials). As a basis for these studies, we are identifying, assessing and following, in each catchment area, population-based cohorts of cases (individuals with an untreated psychotic disorder) and controls (individuals with no history of a psychotic disorder) (figure 2).

In each setting, using methods and infrastructure developed during our feasibility and pilot work, INTREPID I, we will identify, assess and follow at 2 years cohorts of 240 untreated (incident) cases with a psychotic disorder (total=720) and 240 matched controls (total=720). Our inclusion and exclusion criteria for cases are in line with those used in previous studies, including the WHO multicountry studies,^12^ and are purposefully broad to capture heterogeneity and to allow subanalyses by duration of untreated psychosis (table 1).

**Table 1:** Inclusion and exclusion criteria

Inclusion criteria	Exclusion criteria
Cases	
Age 18–64 years	Transient psychotic symptoms resulting from acute intoxication as defined by ICD-10
Currently resident in catchment area (primary residence)	Moderate or severe learning disability, as defined by ICD-10
Presence of ICD-10 psychotic disorder, including substance-induced psychoses	Clinically manifest organic cerebral disorder (eg, infections, parasitic, toxic, cerebrovascular, epilepsy and brain injury), as defined by ICD-10
Not treated with antipsychotic medication for more than one continuous month prior to the start of initial case identification	
Controls	
Age 18–64 years	Past or current ICD-10 psychotic disorder
Currently resident in catchment area (primary residence)	Moderate or severe learning disability, as defined by ICD-10
Same gender as index case	Clinically manifest organic cerebral disorder (eg, infections, parasitic, toxic, cerebrovascular, epilepsy and brain injury), as defined by ICD-10
Within 5 years of age of index case	
Relatives	
Age 18 and above	Insufficient contact with case to provide information on family burden or mental health
Relative or carer of a case who has consented to participate in the current study	

### Sample 1: cases

To estimate incidence, we aim to identify all individuals with an untreated psychotic disorder (cases) within each catchment area. Untreated is defined as never having received treatment with anti-psychotic medication for one continuous month prior to the start of the case-finding period.

In each catchment area, we are using a multipronged approach to case identification. First, using procedures developed in INTREPID I, we have established comprehensive case detection systems by mapping and seeking to engage a comprehensive set of service providers and community key informants who may encounter individuals with psychotic disorders within the catchment area. This includes the professional sector (specialist and generalist services; public, private and third sector), the folk sector (including traditional and religious services) and the popular sector (ie, informal sources of support). Second, we give providers and informants materials developed in our pilot work that detail, using local terms and language, the experiences and behaviours that characterise psychosis. Third, in each catchment area, researchers check with each provider and informant regularly and conduct regular checks of admissions ledgers and registers for in-patient and out-patient services (where these exist), to identify potential cases. In addition, in rural villages in Chennai and Ibadan, field workers visit village meeting points to enquire about potential cases. Potential cases are then screened for inclusion using the Screening Schedule for Psychosis,^51^ an instrument that has been widely used in epidemiological studies of psychoses. Those who screen positive and who meet inclusion criteria are approached and informed consent sought.

Case-finding began on 1 May 2018 and will conclude on 30 April 2020. At the end of the case-finding period, we will conduct leakage studies in each setting to identify possible cases meeting our inclusion criteria who may not have been identified. Each research team will systematically re-check admissions ledgers and registers for in-patient and out-patient services and complete ﬁnal checks with healers and key informants.

All eligible cases identified through the incidence study are invited to participate in the programme. Rates of refusal are documented and basic data (ie, age, gender, area of residence, sector of identification, and where possible ethnicity, religion, duration of untreated psychosis and mode of onset) are collected for those who decline to participate, or who it is not possible to interview, to assess non-response bias.

### Sample 2: controls

Age-matched, sex-matched and neighbourhood-matched controls are recruited to provide indicative population data against which to compare cases in terms of hypothesised risk factors, social outcomes and physical health. We use the Psychosis Screening Questionnaire to collect information on any current or past experiences of psychosis.^80^ In the absence of a readily accessible sampling frame to randomly select potential controls, we map the 10 nearest neighbouring households for each case, listing all residents in these dwellings by sex and age. All potential controls for the case (defined as the same gender and ±5 years of age) are then approached in random order, until an eligible control is identified. When no match is identified the process is repeated. This approach was successfully piloted in all settings.

### Sample 3: relatives and caregivers

We seek consent from each case to approach a close relative or caregiver to participate in the study. We then approach each designated relative to seek his/her consent. The primary purposes of including relatives are to corroborate and extend information from cases (eg, physical health and illness), to collect information on premorbid adjustment, family history of mental disorder and other risk factors, and to collect information on family responses to psychosis, help-seeking and impact (burden) on family.

### Follow-up

All participants will be followed for 2 years. To facilitate this, we collect detailed contact information at baseline (address, telephone number, email address if applicable and service provider details) from each case and control, including details of a relative or friend who can be contacted to trace the individual. In addition, to maintain contact and minimise attrition, we contact participants every 6 months, by telephone or in person, to confirm or update contact details. Based on our pilot work, we expect to re-assess around 80% of cases and controls 2 years after initial identification.

### Sample size

In each setting, we anticipate (based on pilot findings) identifying around 300 untreated incident cases. Of those, given an expected refusal rate of 20% of all eligible cases (based on our pilot work), we anticipate recruiting ~240 cases (total=720), and 240 individually matched controls (total=720). These sample sizes are larger than most previous studies^5 52^ and provide good statistical power to test our hypotheses (ie, >80% at p=0.05). For example: (1) with samples of around 300 untreated incident cases in each setting, we will have over 80% power to detect an incidence rate ratio of 1.5 (or greater) between two areas (eg, urban vs rural), if the incidence rate in the lowest, risk area is 20 per 100,000; (2) with a sample of 240 cases and 240 controls in each setting, we will have over 80% power to detect an OR of 2.0 (or greater) in case–control comparisons when the prevalence of exposure (risk factor) is at least 15% in controls; (3) using gender as an example, with a sample of 192 cases followed at 2 years in each setting, we will have 80% power (or greater) to detect a difference in the proportion of cases with a poor outcome (eg, continuously psychotic) of 0.20 (20%) or greater, when the proportion of men with a poor outcome is 0.40 and the proportion of women is 0.20 (ie, equivalent to an OR of ~2.5).

### Data collection

To test the hypotheses and address the research questions of our four studies, we collect information from cases, relatives and controls at baseline and at a 2-year follow-up. A summary of the measures and the study to which they relate is provided in table 2. All, where necessary, have been translated into local languages and back translated to check equivalence.

**Table 2:** Timing and participants for each measure used in the INTREPID II programme

	Study	Baseline			2-year follow-up		
		Untreated cases (n=720)	Relatives (n=720)	Controls (n=720)	Untreated cases (n~576)	Relatives (n~576)	Controls (n~576)
MRC Sociodemographic Schedule*	1,2,3,4	✓	✓	✓	✓	✓	✓
Personal and Psychiatric History Schedule (PPHS): Baseline^51^*	1,3	✓	✓	–	–	–	–
PPHS: Follow-up^51^*	2,3	–	–	–	✓	✓	–
WHO Life Chart^82^**	2,3	–	–	–	✓	✓	–
Schedules for Clinical Assessment in Neuropsychiatry^83^*	1,2	✓	–	–	✓	–	–
General Assessment of Functioning—Symptoms and Disability scales^84^*	1,2	✓	–	✓	✓	–	✓
WHO Disability Assessment Schedule^85^*	1,2	✓	✓	✓	✓	✓	✓
PANSS^86^*‡	1,2	✓	–	–	✓	–	–
Brief Assessment of Cognition in Schizophrenia^87^	1,2	✓	–	✓	✓	–	✓
Family Interview for Genetic Studies^88^	1,2	✓	✓	✓	–	–	–
Premorbid Adjustment Scale^89^	1,2	✓	✓	–	–	–	–
Alcohol, Smoking and Substance Involvement Screening Test^90^	1,2	✓	–	✓	✓	–	✓
Childhood Trauma Questionnaire^91^	1,2	✓	–	✓	–	–	–
Harvard Trauma Questionnaire^92^	1,2	✓	–	✓	✓	–	✓
List of Threatening Events^93^	1,2	✓	–	✓	✓	–	✓
CIDI§ support networks module	1,2	✓	–	✓	✓	–	✓
Family Burden Interview Schedule^94 95^	3	–	✓	–	–	✓	–
McGill Illness Narrative Interview^96^	3	✓	✓	–	✓	✓	–
WHO STEPS^97^†	4	✓	–	✓	✓	–	✓
Blood pressure	4	✓	–	✓	✓	–	✓
Blood tests	4	✓	–	✓	✓	–	✓
Screen for TB	4	✓	–	✓	✓	–	✓
Medication checklist	4	✓	–	✓	✓	–	✓
Glasgow Antipsychotic Side-effect Scale^98^	4	✓	–	–	✓	–	–
Blood sample for genetics	1	✓	–	✓	–	–	–
GPS coordinates	1,3	✓	–	✓	–	–	–

*Indicates core instruments.

† The WHO STEPwise approach to chronicdisease risk factor surveillance

‡Positive and Negative Syndrome Scale

§Composite International Diagnostic Interview

All those who consent are interviewed and assessed by trained research workers using structured instruments and protocols either at home or at a local clinic. For participants who are in contact with health services, interview data are supplemented with reference to clinical notes, with participants’ consent.

Interviews and assessments are conducted by researchers fluent in the local language. To ensure consistency of methods across settings, all researchers are fully trained using a mixture of online materials and exercises, with feedback, and face to face training, delivered both by the UK team and locally by senior researchers under the supervision of the country principal investigators (PIs). All PIs are experienced psychiatrists with extensive backgrounds in both national and international research. Inter-rater reliability for core instruments that require rater judgement will be tested regularly across settings using video-recorded interviews with cases and controls to ensure that the measures are applied consistently throughout the duration of the programme. Responses will be triangulated with relative reports and, where applicable, clinical records.

### Reliability

All measures will be applied identically, by the same research team, for both cases and controls (where measures apply to both groups). Researchers from across the field settings rated video-taped interviews at study onset and their ratings were compared with gold standard responses developed by the PIs. The mean and range for the proportion of scores that matched the gold standard ratings for each instrument, or were within an acceptable margin, were as follows: Schedules for Clinical Assessment in Neuropsychiatry (SCAN), 87% (85%–88%); Disability Assessment Schedule 88% (85%–92%), Personal and Psychiatric History Schedule 76% (73%–84%), Global Assessment of Function 12.5% (0%–50%). Feedback was provided to the research workers and their ratings will continue to be monitored at repeated intervals throughout the study.

### Analysis plan

We will use standard summary statistics, with indicators of spread and precision as appropriate (eg, crude incidence rates per 100 000 person years, with 95% confidence intervals) to describe the data. We will then use appropriate regression models to compare data between and within settings (eg, Poisson regression for incidence rates and other count data; Cox regression for time-to-event data; logistic regression (including multinomial) for categorical data (eg, course type); and linear regression for continuous data (eg, General Assessment of Functioning score, blood pressure)). In building regression models, we will first fit univariable models, then test for effect modification by core variables (eg, gender, age, setting and time) and finally adjust for putative confounders of each hypothesised association by fitting multivariable models.

Where appropriate, we will use multiple imputations to deal with missing data. In addition, or where assumptions necessary for imputation are not met, we will (re)conduct analyses on participants with complete data only. Where possible, analyses based on imputed data will be presented, with complete data analyses presented as sensitivity analyses in online supplementary materials.

Framework Analysis will be used to analyse qualitative data,^81^ adopting an iterative process of reading and annotating transcripts to identify salient themes, which will form the basis for comparisons between and within settings.

## Ethics and dissemination

Informed consent will be sought from all eligible participants, and participants will be free to withdraw from the study at any time. Capacity to consent will be assessed by trained researchers at the point of seeking consent. If at any point, there is concern for the mental or physical health or welfare of participants, researchers will discuss immediately with the country PI, who will arrange for assessment and referral to the appropriate local mental or other health service, including emergency treatment where necessary.

All data collected will be kept confidential, except with the express consent of the patient to share information with healthcare professionals, or in cases where the participant poses a serious risk either to themselves or to others.

This study has been approved by the ethical review boards of King’s College London (reference number: HR-17/18-5601), London, UK; London School of Hygiene and Tropical Medicine (reference number: 15807), SCARF, Chennai, India; the University of Ibadan, Ibadan, Nigeria; the University of the West Indies, St Augustine, Trinidad; and the North West, North Central, and Eastern Regional Health Authorities of Trinidad.

We will disseminate our findings widely, including through international conferences and publications in international journals, and through locally organised events for service users, service providers and policy makers.

## Patient and public involvement

Patients and members of the public were not involved in the design or conduct of the study. However, the research teams in each study setting are liaising with local service user and family organisations to discuss the interpretation of the findings, to consider potential recommendations arising from the evidence generated and to devise and implement local dissemination plans.

## Ongoing and planned extensions

In addition to enabling us to investigate and test our primary research questions and hypotheses, INTREPID II establishes in each setting platforms and infrastructure for the conduct of other studies. Building on this, several extensions to INTREPID II are ongoing or planned. Four of these are detailed in online supplementary appendix 2 (see Supplementary Materials, online supplementary appendix 2).

## Supplementary Material

Reviewer comments

Author's manuscript
