# Supplementary material for: INTREPID II: protocol for a multistudy programme of research on untreated psychosis in India, Nigeria and Trinidad
Source: BMJ Open. 2020 Jun 21;10(6):e039004. doi: 10.1136/bmjopen-2020-039004 (PMC7311008; doi:10.1136/bmjopen-2020-039004)
Supplement: Supplementary data [file bmjopen-2020-039004supp001.pdf]

Supplementary Materials

STROBE Statement—checklist of items that should be included in reports of observational studies

	Item No	Recommendation	Page No
Title and abstract	1	(a) Indicate the study's design with a commonly used term in the title or the abstract	2
		(b) Provide in the abstract an informative and balanced summary of what was done and what was found	2
Introduction			
Background/rationale	2	Explain the scientific background and rationale for the investigation being reported	4
Objectives	3	State specific objectives, including any prespecified hypotheses	4,5,6,7
Methods			
Study design	4	Present key elements of study design early in the paper	8
Setting	5	Describe the setting, locations, and relevant dates, including periods of recruitment, exposure, follow-up, and data collection	7-8
Participants	6	(a) Cohort study—Give the eligibility criteria, and the sources and methods of selection of participants. Describe methods of follow-up<br>Case-control study—Give the eligibility criteria, and the sources and methods of case ascertainment and control selection. Give the rationale for the choice of cases and controls<br>Cross-sectional study—Give the eligibility criteria, and the sources and methods of selection of participants	8-10, table 1
		(b) Cohort study—For matched studies, give matching criteria and number of exposed and unexposed<br>Case-control study—For matched studies, give matching criteria and the number of controls per case	8-9, table 1
Variables	7	Clearly define all outcomes, exposures, predictors, potential confounders, and effect modifiers. Give diagnostic criteria, if applicable	Table 2
Data sources/ measurement	8*	For each variable of interest, give sources of data and details of methods of assessment (measurement). Describe comparability of assessment methods if there is more than one group	10-11, Table 2
Bias	9	Describe any efforts to address potential sources of bias	10-11

Study size	10	Explain how the study size was arrived at	10
Quantitative variables	11	Explain how quantitative variables were handled in the analyses. If applicable, describe which groupings were chosen and why	N/A (programme protocol covering many research questions)
Statistical methods	12	(a) Describe all statistical methods, including those used to control for confounding	11-12
		(b) Describe any methods used to examine subgroups and interactions	11
		(c) Explain how missing data were addressed	11
		(d) Cohort study—If applicable, explain how loss to follow-up was addressed<br>Case-control study—If applicable, explain how matching of cases and controls was addressed<br>Cross-sectional study—If applicable, describe analytical methods taking account of sampling strategy	11
		(e) Describe any sensitivity analyses	11
Results			
Participants	13*	(a) Report numbers of individuals at each stage of study—eg numbers potentially eligible, examined for eligibility, confirmed eligible, included in the study, completing follow-up, and analysed	N/A (protocol)
		(b) Give reasons for non-participation at each stage	N/A (protocol)
		(c) Consider use of a flow diagram	N/A (protocol)
Descriptive data	14*	(a) Give characteristics of study participants (eg demographic, clinical, social) and information on exposures and potential confounders	N/A (protocol)
		(b) Indicate number of participants with missing data for each variable of interest	N/A (protocol)
		(c) *Cohort study*—Summarise follow-up time (eg, average and total amount)	N/A (protocol)
Outcome data	15*	*Cohort study*—Report numbers of outcome events or summary measures over time	N/A (protocol)
		Case-control study—Report numbers in each exposure category, or summary measures of exposure	N/A (protocol)

		Cross-sectional study—Report numbers of outcome events or summary measures	N/A (protocol)
Main results	16	(*a*) Give unadjusted estimates and, if applicable, confounder-adjusted estimates and their precision (eg, 95% confidence interval). Make clear which confounders were adjusted for and why they were included	N/A (protocol)
		(*b*) Report category boundaries when continuous variables were categorized	N/A (protocol)
		(*c*) If relevant, consider translating estimates of relative risk into absolute risk for a meaningful time period	N/A (protocol)
Other analyses	17	Report other analyses done—eg analyses of subgroups and interactions, and sensitivity analyses	N/A (protocol)
Discussion			
Key results	18	Summarise key results with reference to study objectives	N/A (protocol)
Limitations	19	Discuss limitations of the study, taking into account sources of potential bias or imprecision. Discuss both direction and magnitude of any potential bias	N/A (protocol)
Interpretation	20	Give a cautious overall interpretation of results considering objectives, limitations, multiplicity of analyses, results from similar studies, and other relevant evidence	N/A (protocol)
Generalisability	21	Discuss the generalisability (external validity) of the study results	N/A (protocol)
Other information			
Funding	22	Give the source of funding and the role of the funders for the present study and, if applicable, for the original study on which the present article is based	1

*Give information separately for cases and controls in case-control studies and, if applicable, for exposed and unexposed groups in cohort and cross-sectional studies.

Note: An Explanation and Elaboration article discusses each checklist item and gives methodological background and published examples of transparent reporting. The STROBE checklist is best used in conjunction with this article (freely available on the Web sites of PLoS Medicine at http://www.plosmedicine.org/, Annals of Internal Medicine at http://www.annals.org/, and Epidemiology at http://www.epidem.com/). Information on the STROBE Initiative is available at www.strobe-statement.org.

Appendix 1: Feasibility and Pilot Work

To design and test robust methods to investigate our hypotheses in diverse settings, we conducted extensive feasibility and pilot work in catchment areas in each of the three settings. This consisted of three components. First, we conducted a mapping exercise to identify and engage all professional and folk (traditional) providers and potential key informants within a defined catchment area (approximately half of the area to be covered by INTREPID II), to create a locally tailored case detection system through which to identify and recruit representative samples of cases of psychosis (35). Second, we conducted qualitative research to understand how psychoses are conceptualised locally, in order to facilitate case identification (5). This allowed us to develop locally relevant materials for service providers and key informants identified through our mapping exercise, based on an in-depth understanding of the terminology used to refer to people experiencing psychotic symptoms, the outward manifestations of psychosis that are frequently observed in this context, and patterns of help-seeking. Third, we implemented the methods to be used in INTREPID II for 6-7 months in these catchment areas to assess their feasibility (1-3). This included testing methods for identifying and recruiting age- and sex-matched controls (i.e. non-psychotic individuals) and for following both cases and controls over time.

The pilot project demonstrated that it was possible to identify and recruit both cases and controls through our local detection systems, and to collect extensive data from these participants using the proposed instruments In the process, it established the infrastructure necessary for the INTREPID II programme to conduct larger scale research using these methods in order to test our primary hypotheses. The findings from each of these stages have been described in detail elsewhere (1-3).

References

1. Morgan C, Hibben M, Esan O, John S, Patel V, Weiss HA, et al. Searching for psychosis: INTREPID (1): systems for detecting untreated and first-episode cases of psychosis in diverse settings. Social psychiatry and psychiatric epidemiology. 2015;50(6):879-93.
2. Morgan C, John S, Esan O, Hibben M, Patel V, Weiss H, et al. The incidence of psychoses in diverse settings, INTREPID (2): a feasibility study in India, Nigeria, and Trinidad. Psychological medicine. 2016;46(9):1923-33.
3. Cohen A, Padmavati R, Hibben M, Oyewusi S, John S, Esan O, et al. Concepts of madness in diverse settings: a qualitative study from the INTREPID project. BMC psychiatry. 2016;16(1):388.

Appendix 2: Ongoing and Planned Extensions

In addition to enabling us to investigate and test our primary research questions and hypotheses, INTREPID II establishes in each setting platforms and infrastructure for the conduct of other studies. Building on this, several extensions to INTREPID II are ongoing or planned. Here we highlight four.

(1) Data pooling for international comparisons

A particularly valuable aspect of INTREPID II is that our measures and methods are aligned with previous major research programmes on psychotic disorders in Europe to enable direct comparisons and data pooling to explore variation between countries and populations. For example, secondary hypotheses, regarding incidence rates, initial presentation, and risk factors will be tested by combining INTREPID II data with data from the UK, the Netherlands, France, Spain and Italy collected as part of the AESOP and EU-GEI studies (e.g., that, overall, incidence rates will be lower in India and Nigeria than in Trinidad and northern European countries).

(2) Genetics

Our current understanding of the genetics of psychotic disorders is limited by lack of diversity in the samples used. Most samples comprise individuals of European ancestry. Consequently, when applied to other populations, findings are less applicable. For example, polygenic risk scores (PRS), a measure of the total effects of multiple genes on risk (1), derived from large scale genome-wide association studies, explain far less of the variance in risk when applied in non-European samples (2). INTREPID II, then, provides an opportunity to generate samples from diverse populations that can contribute to global efforts to expand genetic studies to include people of African, Caribbean, and Asian ancestry.

Extending what we originally planned, participating cases and controls will now be invited to provide DNA samples at baseline via blood or saliva samples. Those who provide informed consent will have 10ml blood samples collected in EDTA tubes by a phlebotomy-trained researcher, or 2.5ml of saliva can be provided using Oragene saliva kits. Samples will be shipped to our partner organisations in the USA for DNA extraction and genome-wide association studies (GWAS), with extracted DNA samples then returned to the study settings. By pooling these data with larger datasets, our data will contribute to the generation of PRS that are applicable to our target populations, which we can then use to analyse genetic risk and gene-environment interactions within the INTREPID II cohort.

(3) Spatial Effects

The use of accessible technology to collect GPS coordinates for all participants, service providers and traditional healers provides an opportunity to explore spatial effects – in incidence, risk factors, outcomes, and help-seeking

– at the neighbourhood level. This will allow us to link individual data with ecological data in order to investigate risk and protective factors at the neighbourhood level, as well as facilitating geographical analyses of help-seeking behaviour.

(4) A Global Consortium

INTREPID II will act as a platform for the development of long-term international collaborations to address the lack of evidence on psychotic disorders in the Global South. INTREPID II provides a methodological template for epidemiological research on psychosis across diverse contexts, as exemplified by the establishment of a new research programme in South Africa that uses parallel methods, PSYMAP-ZN (led by Professor Bonginkosi Chiliza, University of KwaZulu-Natal, and Professor Jonathan Burns, University of Exeter). Building on these two programmes, we intend to establish a global consortium for population-based research involving INTREPID II and PSYMAP-ZN researchers and leading psychosis researchers from strategically-chosen settings in more diverse settings across the world – with a particular emphasis on areas where evidence is currently lacking – to extend this research agenda across geographic and disciplinary boundaries. The consortium will involve capacity building, data pooling, knowledge sharing platforms, the development of shared instruments and innovative methods, and will be underpinned by partnerships with service users and carers.

References

1. Consortium IS. Common polygenic variation contributes to risk of schizophrenia and bipolar disorder. Nature. 2009;460(7256):748.
2. Vilhjálmsson BJ, Yang J, Finucane HK, Gusev A, Lindström S, Ripke S, et al. Modeling linkage disequilibrium increases accuracy of polygenic risk scores. The American Journal of Human Genetics. 2015;97(4):576-92.
