# Supplementary material for: INTREPID II: protocol for a multistudy programme of research on untreated psychosis in India, Nigeria and Trinidad
Source: BMJ Open. 2020 Jun 21;10(6):e039004. doi: 10.1136/bmjopen-2020-039004 (PMC7311008; doi:10.1136/bmjopen-2020-039004)
Supplement: Author's manuscript [file bmjopen-2020-039004.draft_revisions.pdf]

BMJ Open

BMJ Open is committed to open peer review. As part of this commitment we make the peer review history of every article we publish publicly available.

When an article is published we post the peer reviewers' comments and the authors' responses online. We also post the versions of the paper that were used during peer review. These are the versions that the peer review comments apply to.

The versions of the paper that follow are the versions that were submitted during the peer review process. They are not the versions of record or the final published versions. They should not be cited or distributed as the published version of this manuscript.

BMJ Open is an open access journal and the full, final, typeset and author-corrected version of record of the manuscript is available on our site with no access controls, subscription charges or pay-per-view fees (http://bmjopen.bmj.com).

If you have any questions on BMJ Open's open peer review process please email info.bmjopen@bmj.com

BMJ Open

INTREPID II: Protocol for a multi-study programme of research on untreated psychosis in India, Nigeria, and Trinidad

Journal:	*BMJ Open*
Manuscript ID	bmjopen-2020-039004
Article Type:	Protocol
Date Submitted by the Author:	31-Mar-2020
Complete List of Authors:	Roberts, Tessa; Institute of Psychiatry Psychology and Neuroscience, Health Service & Population Research department; King's College London, ESRC Centre for Society and Mental Health<br>Gureje, Oye; University of Ibadan, WHO Collaborating Centre for Research and Training in Mental Health, Neuroscience and Substance Abuse, Department of Psychiatry<br>Rangawsamy, Thara; Schizophrenia Research Fondation<br>Hutchinson, Gerard; The University of the West Indies at Saint Augustine Faculty of Medical Sciences<br>Cohen, Alex; Harvard University T H Chan School of Public Health, Department of Epidemiology<br>Weiss, Helen; London School of Hygiene and Tropical Medicine, Epidemiology and Population Health; London School of Hygiene and Tropical Medicine<br>John, Sujit; Schizophrenia Research Foundation<br>Lee Pow, Joni; The University of the West Indies at Saint Augustine Faculty of Medical Sciences<br>Donald, Casswina; The University of the West Indies at Saint Augustine Faculty of Medical Sciences<br>Olley, Bola; University of Ibadan College of Medicine<br>Miguel Esponda, Georgina; Institute of Psychiatry Psychology and Neuroscience, Health Service and Population Research department; King's College London, ESRC Centre for Society and Mental Health<br>Murray, Robin; Institute of Psychiatry Psychology and Neuroscience, Department of Psychosis Studies<br>Morgan, Craig; Institute of Psychiatry Psychology and Neuroscience, Health Service and Population Research department; King's College London, ESRC Centre for Society and Mental Health
Keywords:	Schizophrenia & psychotic disorders < PSYCHIATRY, EPIDEMIOLOGY, International health services < HEALTH SERVICES ADMINISTRATION & MANAGEMENT

SCHOLARONE™
Manuscripts

I, the Submitting Author has the right to grant and does grant on behalf of all authors of the Work (as defined in the below author licence), an exclusive licence and/or a non-exclusive licence for contributions from authors who are: i) UK Crown employees; ii) where BMJ has agreed a CC-BY licence shall apply, and/or iii) in accordance with the terms applicable for US Federal Government officers or employees acting as part of their official duties; on a worldwide, perpetual, irrevocable, royalty-free basis to BMJ Publishing Group Ltd ("BMJ") its licensees and where the relevant Journal is co-owned by BMJ to the co-owners of the Journal, to publish the Work in this journal and any other BMJ products and to exploit all rights, as set out in our licence.

The Submitting Author accepts and understands that any supply made under these terms is made by BMJ to the Submitting Author unless you are acting as an employee on behalf of your employer or a postgraduate student of an affiliated institution which is paying any applicable article publishing charge ("APC") for Open Access articles. Where the Submitting Author wishes to make the Work available on an Open Access basis (and intends to pay the relevant APC), the terms of reuse of such Open Access shall be governed by a Creative Commons licence – details of these licences and which Creative Commons licence will apply to this Work are set out in our licence referred to above.

Other than as permitted in any relevant BMJ Author's Self Archiving Policies, I confirm this Work has not been accepted for publication elsewhere, is not being considered for publication elsewhere and does not duplicate material already published. I confirm all authors consent to publication of this Work and authorise the granting of this licence.

For peer review only

INTREPID II: Protocol for a multi-study programme of research on untreated psychosis in India, Nigeria, and Trinidad

Tessa Roberts[1,7], Oye Gureje[2], Rangaswamy Thara[3], Gerard Hutchinson[4], Alex Cohen[5], Helen A. Weiss[5], Sujit John[3], Joni Lee Pow[4], Casswina Donald[4], Bola Olley[2], Georgina Miguel Esponda[1,7], Robin M. Murray[6], Craig Morgan*[1,7,8].

***Correspondence to**: Professor Craig Morgan, ESRC Centre for Society and Mental Health, Institute of Psychiatry, Psychology & Neuroscience, King's College London. Tel: 020 7848 0351. E-mail: craig.morgan@kcl.ac.uk

[1]Health Service and Population Research Department, Institute of Psychiatry, Psychology & Neuroscience, King's College London, UK; [2] WHO Collaborating Centre for Research and Training in Mental Health, Neuroscience and Substance Abuse, Department of Psychiatry, University of Ibadan, Ibadan, Nigeria; [3]Schizophrenia Research Foundation, Chennai, India; [4]Department of Psychiatry, University of the West Indies, Saint Augustine, Trinidad; [5]MRC Tropical Epidemiology Group, Faculty of Epidemiology and Population Health, London School of Hygiene and Tropical Medicine, London, UK; [6]Psychosis Studies Department, Institute of Psychiatry, Psychology & Neuroscience, King's College London, London, UK; [7]ESRC Centre for Society and Mental Health, King's College London; [8]National Institute for Health Research (NIHR) Mental Health Biomedical Research Centre at South London and Maudsley NHS Foundation Trust and King's College London;

Funding statement

This programme is funded by the UK Medical Research Council (MRC) (MRC Reference: MR/PO25927/1). The authors acknowledge financial support from the National Institute for Health Research (NIHR) Specialist Biomedical Research Centre for Mental Health at South London and Maudsley NHS Foundation Trust and King's College London, and the ESRC Centre for Society and Mental Health at King's College London (ESRC Reference: ES/S012567/1). This programme builds on research funded by the Wellcome Trust (WT094525).

**Abstract Word Count**	250
**Text Body Word Count**	3866
**Tables**	2
**Figures**	2

For peer review only - http://bmjopen.bmj.com/site/about/guidelines.xhtml

Abstract

Background

There are few robust and directly comparable studies of the epidemiology of psychotic disorders in the Global South. INTREPID II is designed to investigate variations in untreated psychotic disorders in the Global South in (1) incidence and presentation, (2) 2-year course and outcome; (3) help-seeking and impact, and (4) physical health.

Methods

INTREPID II is a programme of research incorporating incidence, case-control, and cohort studies of psychoses in contiguous urban and rural areas in India, Nigeria, and Trinidad. In each country, the target samples are 240 untreated cases with a psychotic disorder, 240 age-, sex-, and neighbourhood-matched controls, and 240 relatives or caregivers. Participants will be followed, in the first instance, for 2 years. In each setting, we have developed and are employing comprehensive case-finding methods to ensure cohorts are representative of the target populations. Using methods developed during pilot work, extensive data are being collected at baseline and 2-year follow-up across several domains: clinical, social, help-seeking and impact, and biological.

Ethics and dissemination

Informed consent is sought, and participants are free to withdraw from the study at any time. Participants are referred to mental health services if not already in contact with these and emergency treatment arranged where necessary. All data collected is confidential, except when a participant presents a serious risk to either themselves or others. This programme has been approved by ethical review boards at all participating centres. Findings will be disseminated through international conferences, publications in international journals, and through local events for key stakeholders.

For peer review only - http://bmjopen.bmj.com/site/about/guidelines.xhtml

Article Summary

- Comprehensive case finding methods, building on extensive pilot work, to generate as complete a sample as possible and reduce selection bias,
- Inclusion of population-based, matched controls
- Direct comparability of methods across settings
- Potential trade-offs between cross-setting comparability and local validity
- Use of retrospective self-reports for several factors, which are potentially subject to recall bias and which create challenges in establishing the direction of associations

For peer review only

For peer review only - http://bmjopen.bmj.com/site/about/guidelines.xhtml

INTRODUCTION

Psychotic disorders, such as schizophrenia, affect more than 23 million people worldwide, contribute substantially to the global burden of disease, and are associated with high rates of disability and mortality (1-3). However, there are striking global inequities in our knowledge of psychoses. Over 85% of the world's population lives in Asia, Africa, Latin America, or the Caribbean (referred to here as the Global South)[1], but only a small fraction of research on psychotic disorders is done in these settings (4, 5). This has two implications. First, our knowledge of psychotic disorders, especially of the basic epidemiology, of associated risk factors, and of course and outcome, is incomplete and may be distorted. We do not know whether psychoses manifest, occur, and develop in the same ways around the world. Second, we do not have robust and replicated findings on which to base the development of accessible, humane, and effective services and public health initiatives in low resource settings. Conducting studies in a range of countries and contexts is essential to improve our understanding of the nature of psychotic disorders globally and to provide a much-needed evidence base to inform the development and implementation of effective interventions and services in diverse settings.

We established INTREPID II - the first multi-country study in four decades in the Global South – to extend our knowledge of psychotic disorders in diverse settings. This builds on extensive feasibility and pilot work (5-7) (INTREPID I; see Supplementary Materials, Appendix 1).

AIM, OBJECTIVES, RATIONALE

Our aim is to investigate variability in incidence, presentation, outcome, and impact of untreated psychotic disorders in three diverse countries of the Global South – India, Nigeria, and Trinidad – through four interconnected studies.

Study 1: Incidence, Presentation, and Risk

Objective: To investigate the incidence and presentation of untreated psychotic disorders in each setting and associated risk factors.

Psychotic disorders are highly heterogenous in incidence, presentation, and course and outcome. For example, the incidence of schizophrenia and other psychoses varies markedly across populations and social groups (8, 9). Rates are higher among men (9), in urban areas (10), and in many – but not all – migrant and minority ethnic populations (11). However, little is known about the incidence of psychoses in the Global South, beyond a small number of studies (e.g. (5, 12)), and we cannot assume that findings from the Global North generalise to other

[1]The term Global South refers to countries in Asia, Africa, Latin America and the Caribbean and does not necessarily refer to the geographical south, see e.g. http://www.fc-ssc.org/en/partnership_program/south_south_countries.

For peer review only - http://bmjopen.bmj.com/site/about/guidelines.xhtml

settings. There is tentative evidence, for example, that consistent findings from the Global North, such as the association with urbanicity, may not apply universally (13-15). Further, the phenomenology (i.e., symptom profile) of psychotic disorders is highly varied. Individuals experience a range of symptoms, in various combinations, spanning multiple dimensions, including symptoms of reality distortion (i.e., delusions, hallucinations), thought disturbance, mania, depression, and poverty of affect, speech, and volition. There is some evidence that symptom profiles vary across social and cultural contexts. For example, the Determinants of Outcome of Severe Mental Disorders (DOSMeD) study, a two year cohort study conducted in ten countries by the World Health Organization, found that non-affective acute remitting psychoses (i.e., presentations characterised by rapid onset, symptoms of reality distortion, and quick remission) were around ten times more common in settings in the Global South compared with the Global North (16), but these findings have not been replicated.

There is robust evidence from the Global North implicating an array of factors that likely combine in complex ways to increase risk. These include genetic (17, 18), neurodevelopmental markers (e.g., birth complications, poor premorbid function) (19, 20), exposure to trauma and other social disadvantages (21, 22), migration and minority ethnic status (11, 23), and substance use (24, 25). Further, there is growing evidence that specific risk factors are associated with particular symptoms (18, 26-28). For example, there is evidence of an association between social risk factors and specific symptoms of reality distortion (29-36), i.e. more delusions and hallucinations. It may be, then, that variations in incidence and presentation between settings reflect different population distributions in relevant risks. However, little research has explored these associations in the Global South.

Studying variations in incidence and presentation and associated risks in diverse populations may provide important insights into the aetiology of psychoses and provide a basis for developing public health strategies to reduce the burden of psychotic disorders. In this study, we will test several primary hypotheses on whether variations and associations observed in the Global North hold in more diverse settings.

Study 2: Course and Outcome

Objective: To investigate two-year course and outcome of psychotic disorders and associated factors.

The long-term course and outcome of psychoses following a first episode is highly variable. Evidence from the Global North suggests that, over a period of 5 to 10 years, around half of those with a psychotic disorder recover symptomatically (i.e., are symptom free for a period of 2 or more years) (37-40), but the proportion who achieve both symptom and social recovery is much lower (8-20%) (41), with high levels of enduring unemployment and social isolation (42-47). Several factors are associated with poor symptom and social outcomes, including premorbid difficulties, baseline symptom type (i.e., negative symptoms) and severity, cognition, long duration of untreated psychosis, and persistent substance use (48-50). As with incidence and presentation, it seems that

For peer review only - http://bmjopen.bmj.com/site/about/guidelines.xhtml

course and outcome vary by context. The DOSMeD study (51) and the International Study of Schizophrenia (ISoS) (12) reported better symptom and social outcomes for psychotic disorders in developing (i.e., Global South) vs. developed (i.e., Global North) countries, which has often been attributed to greater family support and community cohesion in more traditional societies. There are, however, several well documented methodological limitations to the DOSMeD and ISoS, not least that the number of countries included from the Global South is small (n, 3). Subsequent research appears to show greater variation between and within countries in the Global South (52).

In this study, we will describe and compare course and outcome at 2 years within and between settings and then test several primary hypotheses on the nature and origins of any observed variations.

Study 3: Help-seeking and Impact

Objective: To investigate: (a) help-seeking; and (b) the impact of psychotic disorders on individuals and families, using a combination of quantitative and qualitative approaches.

Many people with psychotic disorders in the Global South receive no formal (biomedical) treatment or begin treatment well after the critical window when early intervention is most effective (6, 53). Formal care in many countries often falls below minimum quality standards (54), and much of the burden of care falls on families. The use of traditional and religious healing for mental health problems is widespread in both Africa and Asia, even among those who also consult mental health services (55, 56). Such services also exist in the Caribbean, but are more disparate, less specialised, and typically used in addition – rather than as an alternative – to formal health services (5, 6). Practitioners of traditional medicine and faith healing fill a major gap in countries where formal care is scarce (57), but the nature and quality of the care they provide is highly variable (58). Human rights abuses have been widely documented in both traditional healing sites and formal mental health services around the world (59). In part because of this, family members provide a large proportion of care for people with long-standing problems – including severe mental disorders – in the Global South (60). Caring for a relative with a psychotic disorder can have a major physical, emotional and economic impact on families, particularly in households with limited resources (61-63). There is also evidence of high levels of stigma in many countries of the world, including India (64), Nigeria (65), and Trinidad (66).

To plan appropriate services and understand differences in outcomes, further in-depth evidence, both quantitative and qualitative, is needed about how individuals and families respond to psychotic disorders and their needs and experiences, including the treatment they receive, within local contexts. In this study, we will first describe and compare, between and within settings (e.g., by gender, by age, etc.), the types and extent of contacts with formal services and other providers and the impact (i.e., on quality of life) and burden of psychoses for individuals and families. We will then test, using quantitative data, several related primary hypotheses and

For peer review only - http://bmjopen.bmj.com/site/about/guidelines.xhtml

address, using in-depth qualitative data, questions concerning how individuals and families make sense of and respond to psychoses and the impacts on individuals and families.

Study 4: Physical Health

Objective: To investigate the types and prevalence of physical health problems and related biological markers.

In the Global North, those with a psychotic disorder have higher rates of physical health problems and higher rates of all-cause mortality (67), particularly cardiovascular disease and metabolic syndrome (68), which may result from both antipsychotic medication use and lifestyle factors (69-71). Comorbidity of physical and mental health problems is likely to impact negatively on quality of life and recovery (72). Our knowledge of the physical health of people with psychoses in the Global South is much more limited (73), but suggests that there is also a mortality gap compared with the general population and this may be related to similar health problems as in the Global North (74, 75). For example, evidence from India suggests that metabolic syndrome is common (76) and there are rising rates of diabetes and cardiovascular disease in India (77), Nigeria (78), and Trinidad (79). It may also be, however, that the types of physical health problems (e.g., malnutrition; infectious diseases; injury due to accident, violence) in developing countries differ from those common in developed countries.

In this study, we will describe and compare, between and within settings, markers and measures of physical health problems between cases and age- and sex-matched controls, and test hypotheses concerning the nature and origins of variations in physical health within and between settings.

FEASIBILITY AND PILOT WORK

See Appendix 1 in our Supplementary Materials for a description of our feasibility and pilot work.

SETTINGS

INTREPID II is a collaboration between the Schizophrenia Research Foundation (SCARF; Chennai), the University of Ibadan (Nigeria), the University of the West Indies at St Augustine (Trinidad), the London School of Hygiene and Tropical Medicine (UK), and the Institute of Psychiatry, Psychology & Neuroscience, King's College London (UK).

The study settings, in India, Nigeria, and Trinidad, were selected to maximise potential comparisons between sites and with existing datasets. They represent three economically, socially, and culturally diverse areas, on three continents, each undergoing rapid economic and social transformations.

For peer review only - http://bmjopen.bmj.com/site/about/guidelines.xhtml

In each setting, our catchment areas comprise urban and rural areas with total populations of around 500,000 adults aged 18-64 years. In Nigeria, the catchment area comprises three contiguous Local Government Areas in and around the city of Ibadan in Oyo State: Ibadan North East, Ibadan South East, and Ona-Ara (total adult population ~584,000, population density 914 – 18,356 per km^2). In Trinidad, the catchment area comprises the municipalities of Arima, Tunapuna-Piarco, Chaguanas, Port of Spain, San Juan/Laventille, Diego Martin, and Sangre Grande (total adult population ~487,000, population density 82 – 3,090 per km^2). In India the catchment area consists of three contiguous taluks, Chengelpettu, Uthiramerur, and Maduranthakam, located south of Chennai, in the district of Kancheepuram in the state of Tamil Nadu (total adult population ~600,000, population density 361 – 737 per km^2).

METHODS

Overview

INTREPID II comprises four interconnected studies (Figure 1). As a basis for these studies, we are identifying, assessing, and following, in each catchment area, population-based cohorts of cases (individuals with an untreated psychotic disorder) and controls (individuals with no history of a psychotic disorder) (Figure 2).

[Insert Figures 1 and 2]

In each setting, using methods and infrastructure developed during our feasibility and pilot work, INTREPID I, we will identify, assess, and follow at 2 years cohorts of 240 untreated (incident) cases with a psychotic disorder (total, 720) and 240 matched controls (total, 720), using methods developed in INTREPID I. Our inclusion and exclusion criteria for cases are in line with those used in previous studies, including the WHO multi-country studies (12), and are purposefully broad to capture heterogeneity and to allow sub-analyses by duration of untreated psychosis (Table 1).

[Insert Table 1]

Sample (1) Cases

To estimate incidence, we aim to identify all individuals with an untreated psychotic disorder (cases) within each catchment area. Untreated is defined as never having received treatment with anti-psychotic medication for one continuous month prior to the start of the case-finding period.

In each catchment area, we are using a multi-pronged approach to case identification. First, using procedures developed in INTREPID I, we have established comprehensive case detection systems by mapping and seeking to engage a comprehensive set of service providers and community key informants who may encounter

For peer review only - http://bmjopen.bmj.com/site/about/guidelines.xhtml

individuals with psychotic disorders within the catchment area. This includes the professional sector (specialist and generalist services; public, private and third sector), the folk sector (including traditional and religious services), and the popular sector (i.e. informal sources of support). Second, we give providers and informants materials developed in our pilot work that detail, using local terms and language, the experiences and behaviours that characterise psychosis. Third, in each catchment area, researchers check with each provider and informant regularly and conduct regular checks of admissions ledgers and registers for in-patient and out-patient services (where these exist), to identify potential cases. In addition, in rural villages in Chennai and Ibadan, field workers visit village meeting points to enquire about potential cases. Potential cases are then screened for inclusion using the Screening Schedule for Psychosis (51), an instrument that has been widely used in epidemiological studies of psychoses. Those who screen positive and who meet inclusion criteria are approached and informed consent sought.

Case-finding began on 1 May 2018 and will conclude 30 April 2020. At the end of the case-finding period, we will conduct leakage studies in each setting to identify possible cases meeting our inclusion criteria who may not have been identified. Each research team will systematically re-check admissions ledgers and registers for in-patient and out-patient services and complete final checks with healers and key informants.

All eligible cases identified through the incidence study are invited to participate in the programme. Rates of refusal are documented and basic data (i.e. age, gender, area of residence, sector of identification, and where possible ethnicity, religion, duration of untreated psychosis and mode of onset) is collected for those who decline to participate, or who it is not possible to interview, to assess non-response bias.

Sample (2) Controls

Age-, sex- and neighbourhood-matched controls are recruited to provide indicative population data against which to compare cases in terms of hypothesised risk factors, social outcomes, and physical health. We use the Psychosis Screening Questionnaire to collect information on any current or past experiences of psychosis (80). In the absence of a readily accessible sampling frame to randomly select potential controls, we map the ten nearest neighbouring households for each case, listing all residents in these dwellings by sex and age. All potential controls for the case (defined as the same gender and ±5 years of age) are then approached in random order, until an eligible control is identified. When no match is identified the process is repeated. This approach was successfully piloted in all settings.

Sample (3) Relatives and Caregivers

We seek consent from each case to approach a close relative or caregiver to participate in the study. We then approach each designated relative to seek his/her consent. The primary purposes of including relatives are to corroborate and extend information from cases (e.g., physical health and illness), to collect information on

For peer review only - http://bmjopen.bmj.com/site/about/guidelines.xhtml

premorbid adjustment, family history of mental disorder, and other risk factors, and to collect information on family responses to psychosis, help-seeking, and impact (burden) on family.

Follow-up

All participants will be followed at 2 years. To facilitate this, we collect detailed contact information at baseline (address, telephone number, email address if applicable, service provider details) from each case and control, including details of a relative or friend who can be contacted to trace the individual. In addition, to maintain contact and minimise attrition, we contact participants every six months, by telephone or in person, to confirm or update contact details. Based on our pilot work, we expect to re-assess around 80% of cases and controls 2 years after initial identification.

Sample size

In each setting, we anticipate (based on pilot findings) identifying around 300 untreated incident cases. Of those, given an expected refusal rate of 20% of all eligible cases (based on our pilot work), we anticipate recruiting approximately 240 cases (total, 720), and 240 individually matched controls (total, 720). These sample sizes are larger than most previous studies (5, 52) and provide good statistical power to test our hypotheses (i.e., > 80% at p 0.05). For example: (a) with samples of around 300 untreated incident cases in each setting, we will have over 80% power to detect an incidence rate ratio of 1.5 (or greater) between two areas (e.g., urban vs. rural), if the incidence rate in the lowest risk area is 20 per 100,000; (b) with a sample of 240 cases and 240 controls in each setting, we will have over 80% power to detect an odds ratio of 2.0 (or greater) in case-control comparisons when the prevalence of exposure (risk factor) is at least 15% in controls; (c) using gender as an example, with a sample of 192 cases followed at 2 years in each setting, we will have 80% power (or greater) to detect a difference in the proportion of cases with a poor outcome (e.g., continuously psychotic) of 0.20 (20%) or greater, when the proportion of men with a poor outcome is 0.40 and the proportion of women is 0.20 (i.e., equivalent to an odds ratio of ~ 2.5).

Data collection

To test the hypotheses and address the research questions of our 4 studies, we collect information from cases, relatives, and controls at baseline and at 2 year follow up. A summary of the measures and the study to which they relate is provided in Table 2. All, where necessary, have been translated into local languages and back translated to check equivalence.

All those who consent are interviewed and assessed by trained research workers using structured instruments and protocols either at home or at a local clinic. For participants who are in contact with health services, interview data are supplemented with reference to clinical notes, with participants' consent.

For peer review only - http://bmjopen.bmj.com/site/about/guidelines.xhtml

Interviews and assessments are conducted by researchers fluent in the local language. To ensure consistency of methods across settings, all researchers are fully trained using a mixture of online materials and exercises, with feedback, and face to face training, delivered both by the UK team and locally by senior researchers under the supervision of the country principal investigators (PIs). All PIs are experienced psychiatrists with extensive backgrounds in both national and international research. Inter-rater reliability for core instruments that require rater judgement will be tested regularly across settings using video-recorded interviews with cases and controls to ensure that the measures are applied consistently throughout the duration of the programme. Responses will be triangulated with relative reports and, where applicable, clinical records.

[Insert Table 2]

Reliability

All measures will be applied identically, by the same research team, for both cases and controls (where measures apply to both groups). Researchers from across the field settings rated video-taped interviews at study onset and their ratings were compared to gold standard responses developed by the PIs. The mean and range for the proportion of scores that matched the gold standard ratings for each instrument, or were within an acceptable margin, were as follows: Schedules for Clinical Assessment in Neuropsychiatry (SCAN), 87% (85%-88%); Disability Assessment Schedule (DAS) 88% (85%-92%); Personal and Psychiatric History Schedule (PPHS) 76% (73%-84%); Global Assessment of Function 12.5% (0%-50%). Feedback was provided to the research workers and their ratings will continue to be monitored at repeated intervals throughout the study.

Analysis Plan

We will use standard summary statistics, with indicators of spread and precision as appropriate (e.g., crude incidence rates per 100,000 person years, with 95% confidence intervals) to describe the data. We will then use appropriate regression models to compare data between and within settings (e.g., Poisson regression for incidence rates and other count data; Cox regression for time-to-event data; logistic regression (including multinomial) for categorical data [e.g., course type]; and linear regression for continuous data [e.g., GAF score, blood pressure]). In building regression models, we will first fit univariable models, then test for effect modification by core variables (e.g., gender, age, setting, and time), and finally adjust for putative confounders of each hypothesised association by fitting multivariable models.

Where appropriate, we will use multiple imputation to deal with missing data. In addition, or where assumptions necessary for imputation are not met, we will (re) conduct analyses on participants with complete data only. Where possible, analyses based on imputed data will be presented, with complete data analyses presented as sensitivity analyses in supplementary materials.

For peer review only - http://bmjopen.bmj.com/site/about/guidelines.xhtml

Framework Analysis will be used to analyse qualitative data (81), adopting an iterative process of reading and annotating transcripts to identify salient themes, which will form the basis for comparisons between and within settings.

ETHICS AND DISSEMINATION

Informed consent will be sought from all eligible participants, and participants will be free to withdraw from the study at any time. Capacity to consent will be assessed by trained researchers at the point of seeking consent. If at any point, there is concern for the mental or physical health or welfare of participants, researchers will discuss immediately with the country PI, who will arrange for assessment and referral to the appropriate local mental or other health service, including emergency treatment where necessary.

All data collected will be kept confidential, except with the express consent of the patient to share information with health care professionals, or in cases where the participant poses a serious risk either to themselves or to others.

This study has been approved by the ethical review boards of King's College London (reference number: HR-17/18-5601), London, UK; London School of Hygiene and Tropical Medicine (reference number: 15807), the Schizophrenia Research Foundation, Chennai, India; the University of Ibadan, Ibadan, Nigeria; the University of the West Indies, St Augustine, Trinidad; and the North West, North Central, and Eastern Regional Health Authorities of Trinidad.

We will disseminate our findings widely, including through international conferences and publications in international journals, and through locally organised events for service users, service providers, and policy makers.

PATIENT AND PUBLIC INVOLVEMENT

Patients and members of the public were not involved in the design or conduct of the study. However, the research teams in each study setting are liaising with local service user and family organisations to discuss the interpretation of the findings, to consider potential recommendations arising from the evidence generated, and to devise and implement local dissemination plans.

ONGOING AND PLANNED EXTENSIONS

In addition to enabling us to investigate and test our primary research questions and hypotheses, INTREPID II establishes in each setting platforms and infrastructure for the conduct of other studies. Building on this, several

For peer review only - http://bmjopen.bmj.com/site/about/guidelines.xhtml

extensions to INTREPID II are ongoing or planned. Four of these are detailed in Appendix 2 (see Supplementary Materials, Appendix 2).

For peer review only

For peer review only - http://bmjopen.bmj.com/site/about/guidelines.xhtml

REFERENCES

1. Saha S, Chant D, McGrath J. A systematic review of mortality in schizophrenia: is the differential mortality gap worsening over time? Archives of General Psychiatry. 2007;64(10):1123-31.
2. McGrath J, Saha S, Chant D, Welham J. Schizophrenia: a concise overview of incidence, prevalence, and mortality. Epidemiologic Reviews. 2008;30(1):67-76.
3. Charlson FJ, Ferrari AJ, Santomauro DF, Diminic S, Stockings E, Scott JG, et al. Global Epidemiology and Burden of Schizophrenia: Findings From the Global Burden of Disease Study 2016. Schizophrenia Bulletin. 2018.
4. McGrath J, Saha S, Welham J, El Saadi O, MacCauley C, Chant D. A systematic review of the incidence of schizophrenia: the distribution of rates and the influence of sex, urbanicity, migrant status and methodology. BMC Medicine. 2004;2(1):13.
5. Morgan C, John S, Esan O, Hibben M, Patel V, Weiss H, et al. The incidence of psychoses in diverse settings, INTREPID (2): a feasibility study in India, Nigeria, and Trinidad. Psychological Medicine. 2016;46(9):1923-33.
6. Morgan C, Hibben M, Esan O, John S, Patel V, Weiss HA, et al. Searching for psychosis: INTREPID (1): systems for detecting untreated and first-episode cases of psychosis in diverse settings. Social Psychiatry and Psychiatric Epidemiology. 2015;50(6):879-93.
7. Cohen A, Padmavati R, Hibben M, Oyewusi S, John S, Esan O, et al. Concepts of madness in diverse settings: a qualitative study from the INTREPID project. BMC Psychiatry. 2016;16(1):388.
8. Jongsma HE, Turner C, Kirkbride JB, Jones PB. International incidence of psychotic disorders, 2002–17: a systematic review and meta-analysis. The Lancet Public Health. 2019;4(5):e229-e44.
9. McGrath JJ. The surprisingly rich contours of schizophrenia epidemiology. Archives of General Psychiatry. 2007;64(1):14-6.
10. March D, Hatch SL, Morgan C, Kirkbride JB, Bresnahan M, Fearon P, et al. Psychosis and place. Epidemiologic Reviews. 2008;30(1):84-100.
11. Morgan C, Charalambides M, Hutchinson G, Murray RM. Migration, ethnicity, and psychosis: toward a sociodevelopmental model. Schizophrenia Bulletin. 2010;36(4):655-64.
12. Hopper K, Harrison G, Janca A, Norman Sartorius M. Recovery from schizophrenia: An international perspective: A report from the WHO Collaborative Project, the international study of schizophrenia: Oxford University Press; 2007.
13. Menezes PR, Scazufca M, Busatto GF, Coutinho LM, McGUIRE PK, Murray RM. Incidence of first-contact psychosis in Sao Paulo, Brazil. The British Journal of Psychiatry. 2007;191(51):s102-s6.
14. Jongsma HE, Gayer-Anderson C, Lasalvia A, Quattrone D, Mulè A, Szöke A, et al. Treated incidence of psychotic disorders in the multinational EU-GEI study. JAMA Psychiatry. 2018;75(1):36-46.
15. DeVylder JE, Kelleher I, Lalane M, Oh H, Link BG, Koyanagi A. Association of urbanicity with psychosis in low-and middle-income countries. JAMA Psychiatry. 2018;75(7):678-86.

For peer review only - http://bmjopen.bmj.com/site/about/guidelines.xhtml

16. Susser E, Wanderling J. Epidemiology of nonaffective acute remitting psychosis vs schizophrenia. Arch Gen Psychiatry. 1994;51:294-301.

17. Van Os J, Marcelis M, Sham P, Jones P, Gilvarry K, Murray R. Psychopathological syndromes and familial morbid risk of psychosis. The British Journal of Psychiatry. 1997;170(3):241-6.

18. Peralta V, Cuesta MJ. The relationship between syndromes of the psychotic illness and familial liability to schizophrenia and major mood disorders. Schizophrenia Research. 2007;91(1-3):200-9.

19. Tarbox SI, Addington J, Cadenhead KS, Cannon TD, Cornblatt BA, Perkins DO, et al. Premorbid functional development and conversion to psychosis in clinical high-risk youths. Development and Psychopathology. 2013;25(4pt1):1171-86.

20. Lyngberg K, Buchy L, Liu L, Perkins D, Woods S, Addington J. Patterns of premorbid functioning in individuals at clinical high risk of psychosis. Schizophrenia Research. 2015;169(1-3):209-13.

21. Morgan C, Gayer-Anderson C. Childhood adversities and psychosis: evidence, challenges, implications. World Psychiatry. 2016;15(2):93-102.

22. Read J, van Os J, Morrison AP, Ross CA. Childhood trauma, psychosis and schizophrenia: a literature review with theoretical and clinical implications. Acta Psychiatrica Scandinavica. 2005;112(5):330-50.

23. Selten J-P, van der Ven E, Termorshuizen F. Migration and psychosis: a meta-analysis of incidence studies. Psychological Medicine. 2019:1-11.

24. Di Forti M, Marconi A, Carra E, Fraietta S, Trotta A, Bonomo M, et al. Proportion of patients in south London with first-episode psychosis attributable to use of high potency cannabis: a case-control study. The Lancet Psychiatry. 2015;2(3):233-8.

25. Marconi A, Di Forti M, Lewis CM, Murray RM, Vassos E. Meta-analysis of the association between the level of cannabis use and risk of psychosis. Schizophrenia Bulletin. 2016;42(5):1262-9.

26. van Nierop M, Viechtbauer W, Gunther N, Van Zelst C, De Graaf R, Ten Have M, et al. Childhood trauma is associated with a specific admixture of affective, anxiety, and psychosis symptoms cutting across traditional diagnostic boundaries. Psychological Medicine. 2015;45(6):1277-88.

27. Van Os J, Jones P, Sham P, Bebbington P, Murray R. Risk factors for onset and persistence of psychosis. Social Psychiatry and Psychiatric Epidemiology. 1998;33(12):596-605.

28. Allardyce J, McCreadie RG, Morrison G, van Os J. Do symptom dimensions or categorical diagnoses best discriminate between known risk factors for psychosis? SocialPsychiatry and Psychiatric Epidemiology. 2007;42(6):429-37.

29. Demjaha A, Morgan K, Morgan C, Dazzan P, Landau S, Dean K, et al., editors. Symptom dimensions and ethnicity in the AESOP first onset psychosis study. Schizophrenia Research; 2006: ELSEVIER SCIENCE BV PO BOX 211, 1000 AE AMSTERDAM, NETHERLANDS.

30. Berg AO, Andreassen OA, Aminoff SR, Romm KL, Hauff E, Melle I. The impact of immigration and visible minority status on psychosis symptom profile. Social Psychiatry and Psychiatric Epidemiology. 2014;49(11):1747-57.

For peer review only - http://bmjopen.bmj.com/site/about/guidelines.xhtml

31. van der Ven E, Bourque F, Joober R, Selten J-P, Malla AK. Comparing the clinical presentation of first-episode psychosis across different migrant and ethnic minority groups in Montreal, Quebec. The Canadian Journal of Psychiatry. 2012;57(5):300-8.

32. Oher F, Demjaha A, Jackson D, Morgan C, Dazzan P, Morgan K, et al. The effect of the environment on symptom dimensions in the first episode of psychosis: a multilevel study. Psychological Medicine. 2014;44(11):2419-30.

33. Demjaha A, MacCabe JH, Murray RM. How genes and environmental factors determine the different neurodevelopmental trajectories of schizophrenia and bipolar disorder. Schizophrenia Bulletin. 2011;38(2):209-14.

34. Demjaha A, Morgan K, Morgan C, Landau S, Dean K, Reichenberg A, et al. Combining dimensional and categorical representation of psychosis: the way forward for DSM-V and ICD-11? Psychological Medicine. 2009;39(12):1943-55.

35. Bentall RP, de Sousa P, Varese F, Wickham S, Sitko K, Haarmans M, et al. From adversity to psychosis: pathways and mechanisms from specific adversities to specific symptoms. Social psychiatry and Psychiatric Epidemiology. 2014;49(7):1011-22.

36. Quattrone D, Di Forti M, Gayer-Anderson C, Ferraro L, Jongsma HE, Tripoli G, et al. Transdiagnostic dimensions of psychopathology at first episode psychosis: findings from the multinational EU-GEI study. Psychological Medicine. 2019;49(8):1378-91.

37. Morgan C, Lappin J, Heslin M, Donoghue K, Lomas B, Reininghaus U, et al. Reappraising the long-term course and outcome of psychotic disorders: the AESOP-10 study. Psychological Medicine. 2014;44(13):2713-26.

38. Jobe TH, Harrow M. Long-term outcome of patients with schizophrenia: a review. The Canadian Journal of Psychiatry. 2005;50(14):892-900.

39. Harrow M, Grossman LS, Jobe TH, Herbener ES. Do patients with schizophrenia ever show periods of recovery? A 15-year multi-follow-up study. Schizophrenia Bulletin. 2005;31(3):723-34.

40. Harrison G, Hopper K, Craig T, Laska E, Siegel C, Wanderling J, et al. Recovery from psychotic illness: a 15-and 25-year international follow-up study. The British Journal of Psychiatry. 2001;178(6):506-17.

41. Jääskeläinen E, Juola P, Hirvonen N, McGrath JJ, Saha S, Isohanni M, et al. A systematic review and meta-analysis of recovery in schizophrenia. Schizophrenia Bulletin. 2012;39(6):1296-306.

42. Stain HJ, Galletly CA, Clark S, Wilson J, Killen EA, Anthes L, et al. Understanding the social costs of psychosis: the experience of adults affected by psychosis identified within the second Australian National Survey of Psychosis. Australian & New Zealand Journal of Psychiatry. 2012;46(9):879-89.

43. Killaspy H, White S, Lalvani N, Berg R, Thachil A, Kallumpuram S, et al. The impact of psychosis on social inclusion and associated factors. International Journal of Social Psychiatry. 2014;60(2):148-54.

44. Gayer-Anderson C, Morgan C. Social networks, support and early psychosis: a systematic review. Epidemiology and Psychiatric Sciences. 2013;22(2):131-46.

For peer review only

45. Waghorn G, Saha S, Harvey C, Morgan VA, Waterreus A, Bush R, et al. 'Earning and learning'in those with psychotic disorders: The second Australian national survey of psychosis. Australian & New Zealand Journal of Psychiatry. 2012;46(8):774-85.

46. Kooyman I, Dean K, Harvey S, Walsh E. Outcomes of public concern in schizophrenia. The British Journal of Psychiatry. 2007;191(S50):s29-s36.

47. Marwaha S, Johnson S, Bebbington P, Stafford M, Angermeyer MC, Brugha T, et al. Rates and correlates of employment in people with schizophrenia in the UK, France and Germany. The British Journal of Psychiatry. 2007;191(1):30-7.

48. Menezes N, Arenovich T, Zipursky R. A systematic review of longitudinal outcome studies of first-episode psychosis. Psychological Medicine. 2006;36(10):1349-62.

49. Alvarez-Jimenez M, Priede A, Hetrick S, Bendall S, Killackey E, Parker A, et al. Risk factors for relapse following treatment for first episode psychosis: a systematic review and meta-analysis of longitudinal studies. SchizophreniaResearch. 2012;139(1-3):116-28.

50. Díaz-Caneja CM, Pina-Camacho L, Rodríguez-Quiroga A, Fraguas D, Parellada M, Arango C. Predictors of outcome in early-onset psychosis: a systematic review. npj Schizophrenia. 2015;1:14005.

51. Jablensky A, Sartorius N, Ernberg G, Anker M, Korten A, Cooper JE, et al. Schizophrenia: manifestations, incidence and course in different cultures A World Health Organization Ten-Country Study. Psychological Medicine Monograph Supplement. 1992;20:1-97.

52. Cohen A, Patel V, Thara R, Gureje O. Questioning an axiom: better prognosis for schizophrenia in the developing world? Schizophrenia Bulletin. 2007;34(2):229-44.

53. Demyttenaere K, Bruffaerts R, Posada-Villa J, Gasquet I, Kovess V, Lepine J, et al. Prevalence, severity, and unmet need for treatment of mental disorders in the World Health Organization World Mental Health Surveys. JAMA. 2004;291(21):2581-90.

54. Asher L, Fekadu A, Hanlon C. Global mental health and schizophrenia. Current Opinion in Psychiatry. 2018;31(3):193-9.

55. Burns JK, Tomita A. Traditional and religious healers in the pathway to care for people with mental disorders in Africa: a systematic review and meta-analysis. Social Psychiatry and Psychiatric Epidemiology. 2015;50(6):867-77.

56. Thirthalli J, Zhou L, Kumar K, Gao J, Vaid H, Liu H, et al. Traditional, complementary, and alternative medicine approaches to mental health care and psychological wellbeing in India and China. The Lancet Psychiatry. 2016;3(7):660-72.

57. Gureje O, Nortje G, Makanjuola V, Oladeji BD, Seedat S, Jenkins R. The role of global traditional and complementary systems of medicine in the treatment of mental health disorders. The Lancet Psychiatry. 2015;2(2):168-77.

58. Nortje G, Oladeji B, Gureje O, Seedat S. Effectiveness of traditional healers in treating mental disorders: a systematic review. The Lancet Psychiatry. 2016;3(2):154-70.

For peer review only - http://bmjopen.bmj.com/site/about/guidelines.xhtml

59. Drew N, Funk M, Tang S, Lamichhane J, Chávez E, Katontoka S, et al. Human rights violations of people with mental and psychosocial disabilities: an unresolved global crisis. The Lancet. 2011;378(9803):1664-75.
60. Thrush A, Hyder A. The neglected burden of caregiving in low-and middle-income countries. Disability and Health Journal. 2014;7(3):262-72.
61. Awad AG, Voruganti LN. The burden of schizophrenia on caregivers. Pharmacoeconomics. 2008;26(2):149-62.
62. Caqueo-Urízar A, Gutiérrez-Maldonado J, Miranda-Castillo C. Quality of life in caregivers of patients with schizophrenia: a literature review. Health and Quality of Life Outcomes. 2009;7(1):84.
63. Chan SW-c. Global perspective of burden of family caregivers for persons with schizophrenia. Archives of Psychiatric Nursing. 2011;25(5):339-49.
64. Loganathan S, Murthy SR. Experiences of stigma and discrimination endured by people suffering from schizophrenia. Indian Journal of Psychiatry. 2008;50(1):39.
65. Gureje O, Lasebikan VO, Ephraim-Oluwanuga O, Olley BO, Kola L. Community study of knowledge of and attitude to mental illness in Nigeria. The British Journal of Psychiatry. 2005;186(5):436-41.
66. Hutchinson G, Neehall JE, Simeon DT, Littlewood R. Perceptions about mental illness among pre-clinical medical students in Trinidad & Tobago. The West Indian Medical Journal. 1999;48(2):81-4.
67. Reininghaus U, Dutta R, Dazzan P, Doody GA, Fearon P, Lappin J, et al. Mortality in schizophrenia and other psychoses: a 10-year follow-up of the ÆSOP first-episode cohort. Schizophrenia Bulletin. 2014;41(3):664-73.
68. Doherty AM, Gaughran F. The interface of physical and mental health. Social psychiatry and psychiatric epidemiology. 2014;49(5):673-82.
69. Meyer JM, Stahl SM. The metabolic syndrome and schizophrenia. Acta Psychiatrica Scandinavica. 2009;119(1):4-14.
70. HERT M, Schreurs V, Vancampfort D, Winkel R. Metabolic syndrome in people with schizophrenia: a review. World Psychiatry. 2009;8(1):15-22.
71. Mitchell AJ, Vancampfort D, De Herdt A, Yu W, De Hert M. Is the prevalence of metabolic syndrome and metabolic abnormalities increased in early schizophrenia? A comparative meta-analysis of first episode, untreated and treated patients. Schizophrenia Bulletin. 2012;39(2):295-305.
72. Firth J, Siddiqi N, Koyanagi A, Siskind D, Rosenbaum S, Galletly C, et al. The Lancet Psychiatry Commission: a blueprint for protecting physical health in people with mental illness. The Lancet Psychiatry. 2019;6(8):675-712.
73. Hjorthøj C, Stürup AE, McGrath JJ, Nordentoft M. Years of potential life lost and life expectancy in schizophrenia: a systematic review and meta-analysis. The Lancet Psychiatry. 2017;4(4):295-301.
74. Fekadu A, Medhin G, Kebede D, Alem A, Cleare AJ, Prince M, et al. Excess mortality in severe mental illness: 10-year population-based cohort study in rural Ethiopia. The British Journal of Psychiatry. 2015;206(4):289-96.

For peer review only - http://bmjopen.bmj.com/site/about/guidelines.xhtml

75. Lêng CH, Chou MH, Lin S-H, Yang YK, Wang J-D. Estimation of life expectancy, loss-of-life expectancy, and lifetime healthcare expenditures for schizophrenia in Taiwan. Schizophrenia Research. 2016;171(1-3):97-102.

76. Padmavati R. Metabolic syndrome, serious mental illnesses & lifestyle. The Indian Journal of Medical Research. 2016;143(4):395.

77. Mohan V, Sandeep S, Deepa R, Shah B, Varghese C. Epidemiology of type 2 diabetes: Indian scenario. Indian Journal of Medical Research. 2007;125(3):217.

78. Azevedo M, Alla S. Diabetes in sub-saharan Africa: kenya, mali, mozambique, Nigeria, South Africa and zambia. International Journal of Diabetes in Developing Countries. 2008;28(4):101.

79. Joseph L. An assessment of knowledge, attitude, beliefs and risk perception of type 2 diabetes mellitus among the adult population of Trinidad and Tobago. 2016.

80. Bebbington P, Nayani T. The psychosis screening questionnaire. International Journal of Methods in Psychiatric Research. 1995.

81. Gale NK, Heath G, Cameron E, Rashid S, Redwood S. Using the framework method for the analysis of qualitative data in multi-disciplinary health research. BMC Medical Research Methodology. 2013;13(1):117.

For peer review only

For peer review only - http://bmjopen.bmj.com/site/about/guidelines.xhtml

Author Contributions

TR contributes to the overall coordination of the programme and drafted and revised the manuscript, based on the funded grant proposal written by CM, OG, RT, GH, HW, AC, and RM to the UK Medical Research Council.

RT, OG, GH contributed to the design the programme, lead the programme in India, Nigeria, and Trinidad, respectively, and contributed to review and revision of the manuscript.

AC, HW, RM contributed to the design of the programme and to review and revision of the manuscript.

GME contributes to the overall coordination of the programme and contributed to review and revision of the manuscript.

SJ, BO, JLP, CD coordinate the programme in India (SL), Nigeria (BO) and Trinidad (JLP, CO) and contributed to review and revision of the manuscript.

CM led the design of the programme and the study methods, leads the programme, contributed to drafting and revising the manuscript, and provided guidance and supervision throughout the preparation of the manuscript.

Competing Interests Statement

RM has received payment for lectures from Janssen, Sunovian, Otsuka, Lundbeck, Angelini, and Rekordati.

Data Statement

The current article is a protocol for an ongoing study. When data are available, they will be shared upon reasonable request after submitting a synopsis of any proposed analyses to the study coordinator and receiving approval from the study PIs.

Acknowledgements

The authors wish to thank the research teams at the University of Ibadan, the Schizophrenia Research Foundation, and the University of the West Indies, for their ongoing work on this programme.

For peer review only - http://bmjopen.bmj.com/site/about/guidelines.xhtml

Table 1. Inclusion and exclusion criteria.

	Inclusion criteria	Exclusion criteria
Cases	• Age 18 to 64 years<br>• Currently resident in catchment area (primary residence)<br>• Presence of ICD-10 psychotic disorder, including substance-induced psychoses<br>• Not treated with antipsychotic medication for more than one continuous month prior to the start of initial case identification	• Transient psychotic symptoms resulting from acute intoxication as defined by ICD-10<br>• Moderate or severe learning disability, as defined by ICD-10<br>• Clinically manifest organic cerebral disorder (e.g. infections, parasitic, toxic, cerebrovascular, epilepsy, brain injury), as defined by ICD-10
Controls	• Age 18 to 64 years<br>• Currently resident in catchment area (primary residence)<br>• Same gender as index case<br>• Within 5 years of age of index case	• Past or current ICD-10 psychotic disorder<br>• Moderate or severe learning disability, as defined by ICD-10<br>• Clinically manifest organic cerebral disorder (e.g. infections, parasitic, toxic, cerebrovascular, epilepsy, brain injury), as defined by ICD-10
Relatives	• Age 18 and above<br>• Relative or carer of a case who has consented to participate in the current study	• Insufficient contact with case to provide information on family burden or mental health

For peer review only

Table 2. Timing and participants for each measure used in the INTREPID II programme.

	**Study**	**Baseline**			**2-year follow-up**		
		Untreated cases (n, 720)	Relatives (n, 720)	Controls (n, 720)	Untreated cases (n, ~ 576)	Relatives (n, ~ 576)	Controls (n, ~ 576)
MRC Sociodemographic Schedule*	1,2,3,4	✓	✓	✓	✓	✓	✓
Personal and Psychiatric History Schedule (PPHS): Baseline* (16)	1,3	✓	✓	-	-	-	-
Personal and Psychiatric History Schedule (PPHS): Follow-up* (16)	2,3	-	-	-	✓	✓	-
WHO Life Chart* (84)	2,3	-	-	-	✓	✓	-
Schedules for Clinical Assessment in Neuropsychiatry (SCAN)* (85)	1,2	✓	-	-	✓	-	-
General Assessment of Functioning (GAF) - Symptoms & Disability scales* (86)	1,2	✓	-	✓	✓	-	✓
WHO Disability Assessment Schedule (DAS)* (87)	1,2	✓	✓	✓	✓	✓	✓
PANSS* (88)	1,2	✓	-	-	✓	-	-
Brief Assessment of Cognition in Schizophrenia (BACS) (89)	1,2	✓	-	✓	✓	-	✓
Family Interview for Genetic Studies (FIGS) (90)	1,2	✓	✓	✓	-	-	-
Premorbid Adjustment Scale (PAS) (91)	1,2	✓	✓	-	-	-	-
Alcohol, Smoking and Substance Involvement Screening Test (ASSIST) (92)	1,2	✓	-	✓	✓	-	✓
Childhood Trauma Questionnaire (CTQ) (93)	1,2	✓	-	✓	-	-	-
Harvard Trauma Questionnaire (HTQ) (94)	1,2	✓	-	✓	✓	-	✓

For peer review only

List of Threatening Events (LoTE) (95)	1,2	✓	-	✓	✓	-	✓
CIDI support networks module	1,2	✓	-	✓	✓	-	✓
Family Burden Interview Schedule (FBIS) (96, 97)	3	-	✓	-	-	✓	-
McGill Illness Narrative Interview (MINI) (98)	3	✓	✓	-	✓	✓	-
WHO STEPS (99)	4	✓	-	✓	✓	-	✓
Blood pressure	4	✓	-	✓	✓	-	✓
Blood tests	4	✓	-	✓	✓	-	✓
Screen for TB	4	✓	-	✓	✓	-	✓
Medication checklist	4	✓	-	✓	✓	-	✓
Glasgow Antipsychotic Side-effect Scale (100)	4	✓	-	-	✓	-	-
Blood sample for genetics	1	✓	-	✓	-	-	-
GPS coordinates	1,3	✓	-	✓	-	-	-

* Indicates core instruments

For peer review only

Study	Baseline<br>720 cases; 720 controls	2 Year Follow-Up<br>~ 576 cases; ~ 576 controls
(1) Incidence and presentation	X	-
(2) Course and outcome	X	X
(3) Help-Seeking and Impact	X	X
(4) Physical Health	X	X

Figure 1. Structure of INTREPID II.

For peer review only

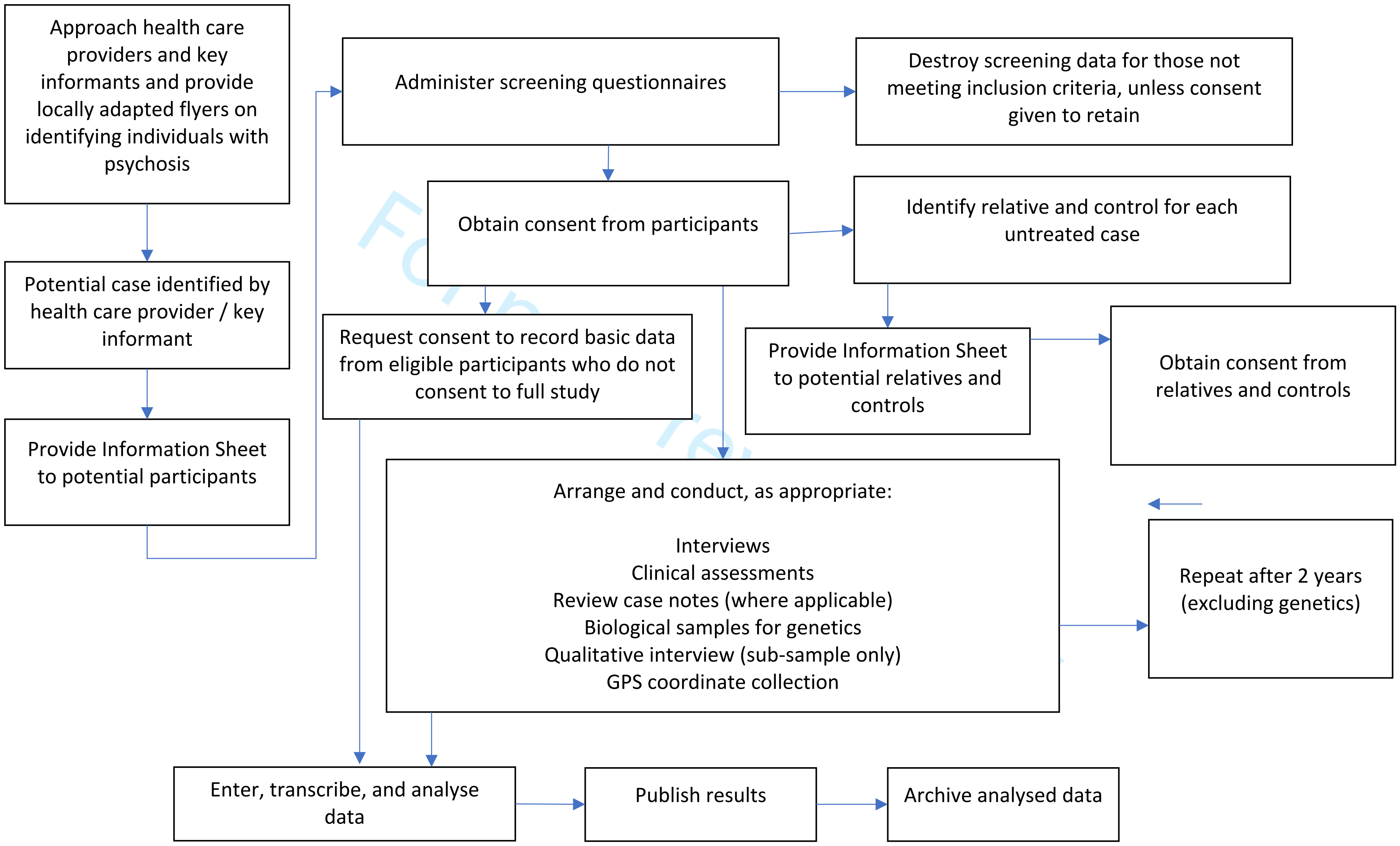

Figure 2. Summary of methodology.

Supplementary Materials

STROBE Statement—checklist of items that should be included in reports of observational studies

	Item No	Recommendation	Page No
Title and abstract	1	(a) Indicate the study's design with a commonly used term in the title or the abstract	2
		(b) Provide in the abstract an informative and balanced summary of what was done and what was found	2
Introduction			
Background/rationale	2	Explain the scientific background and rationale for the investigation being reported	4
Objectives	3	State specific objectives, including any prespecified hypotheses	4,5,6,7
Methods			
Study design	4	Present key elements of study design early in the paper	8
Setting	5	Describe the setting, locations, and relevant dates, including periods of recruitment, exposure, follow-up, and data collection	7-8
Participants	6	(a) Cohort study—Give the eligibility criteria, and the sources and methods of selection of participants. Describe methods of follow-up<br>Case-control study—Give the eligibility criteria, and the sources and methods of case ascertainment and control selection. Give the rationale for the choice of cases and controls<br>Cross-sectional study—Give the eligibility criteria, and the sources and methods of selection of participants	8-10, table 1
		(b) Cohort study—For matched studies, give matching criteria and number of exposed and unexposed<br>Case-control study—For matched studies, give matching criteria and the number of controls per case	8-9, table 1
Variables	7	Clearly define all outcomes, exposures, predictors, potential confounders, and effect modifiers. Give diagnostic criteria, if applicable	Table 2
Data sources/ measurement	8*	For each variable of interest, give sources of data and details of methods of assessment (measurement). Describe comparability of assessment methods if there is more than one group	10-11, Table 2
Bias	9	Describe any efforts to address potential sources of bias	10-11

For peer review only

Study size	10	Explain how the study size was arrived at	10
Quantitative variables	11	Explain how quantitative variables were handled in the analyses. If applicable, describe which groupings were chosen and why	N/A (programme protocol covering many research questions)
Statistical methods	12	(a) Describe all statistical methods, including those used to control for confounding	11-12
		(b) Describe any methods used to examine subgroups and interactions	11
		(c) Explain how missing data were addressed	11
		(d) Cohort study—If applicable, explain how loss to follow-up was addressed<br>Case-control study—If applicable, explain how matching of cases and controls was addressed<br>Cross-sectional study—If applicable, describe analytical methods taking account of sampling strategy	11
		(e) Describe any sensitivity analyses	11
Results			
Participants	13*	(a) Report numbers of individuals at each stage of study—eg numbers potentially eligible, examined for eligibility, confirmed eligible, included in the study, completing follow-up, and analysed	N/A (protocol)
		(b) Give reasons for non-participation at each stage	N/A (protocol)
		(c) Consider use of a flow diagram	N/A (protocol)
Descriptive data	14*	(a) Give characteristics of study participants (eg demographic, clinical, social) and information on exposures and potential confounders	N/A (protocol)
		(b) Indicate number of participants with missing data for each variable of interest	N/A (protocol)
		(c) *Cohort study*—Summarise follow-up time (eg, average and total amount)	N/A (protocol)
Outcome data	15*	*Cohort study*—Report numbers of outcome events or summary measures over time	N/A (protocol)
		Case-control study—Report numbers in each exposure category, or summary measures of exposure	N/A (protocol)

		Cross-sectional study—Report numbers of outcome events or summary measures	N/A (protocol)
Main results	16	(*a*) Give unadjusted estimates and, if applicable, confounder-adjusted estimates and their precision (eg, 95% confidence interval). Make clear which confounders were adjusted for and why they were included	N/A (protocol)
		(*b*) Report category boundaries when continuous variables were categorized	N/A (protocol)
		(*c*) If relevant, consider translating estimates of relative risk into absolute risk for a meaningful time period	N/A (protocol)
Other analyses	17	Report other analyses done—eg analyses of subgroups and interactions, and sensitivity analyses	N/A (protocol)
Discussion			
Key results	18	Summarise key results with reference to study objectives	N/A (protocol)
Limitations	19	Discuss limitations of the study, taking into account sources of potential bias or imprecision. Discuss both direction and magnitude of any potential bias	N/A (protocol)
Interpretation	20	Give a cautious overall interpretation of results considering objectives, limitations, multiplicity of analyses, results from similar studies, and other relevant evidence	N/A (protocol)
Generalisability	21	Discuss the generalisability (external validity) of the study results	N/A (protocol)
Other information			
Funding	22	Give the source of funding and the role of the funders for the present study and, if applicable, for the original study on which the present article is based	1

*Give information separately for cases and controls in case-control studies and, if applicable, for exposed and unexposed groups in cohort and cross-sectional studies.

Note: An Explanation and Elaboration article discusses each checklist item and gives methodological background and published examples of transparent reporting. The STROBE checklist is best used in conjunction with this article (freely available on the Web sites of PLoS Medicine at http://www.plosmedicine.org/, Annals of Internal Medicine at http://www.annals.org/, and Epidemiology at http://www.epidem.com/). Information on the STROBE Initiative is available at www.strobe-statement.org.

For peer review only

Appendix 1: Feasibility and Pilot Work

To design and test robust methods to investigate our hypotheses in diverse settings, we conducted extensive feasibility and pilot work in catchment areas in each of the three settings. This consisted of three components. First, we conducted a mapping exercise to identify and engage all professional and folk (traditional) providers and potential key informants within a defined catchment area (approximately half of the area to be covered by INTREPID II), to create a locally tailored case detection system through which to identify and recruit representative samples of cases of psychosis (35). Second, we conducted qualitative research to understand how psychoses are conceptualised locally, in order to facilitate case identification (5). This allowed us to develop locally relevant materials for service providers and key informants identified through our mapping exercise, based on an in-depth understanding of the terminology used to refer to people experiencing psychotic symptoms, the outward manifestations of psychosis that are frequently observed in this context, and patterns of help-seeking. Third, we implemented the methods to be used in INTREPID II for 6-7 months in these catchment areas to assess their feasibility (1-3). This included testing methods for identifying and recruiting age- and sex-matched controls (i.e. non-psychotic individuals) and for following both cases and controls over time.

The pilot project demonstrated that it was possible to identify and recruit both cases and controls through our local detection systems, and to collect extensive data from these participants using the proposed instruments In the process, it established the infrastructure necessary for the INTREPID II programme to conduct larger scale research using these methods in order to test our primary hypotheses. The findings from each of these stages have been described in detail elsewhere (1-3).

References

1. Morgan C, Hibben M, Esan O, John S, Patel V, Weiss HA, et al. Searching for psychosis: INTREPID (1): systems for detecting untreated and first-episode cases of psychosis in diverse settings. Social psychiatry and psychiatric epidemiology. 2015;50(6):879-93.
2. Morgan C, John S, Esan O, Hibben M, Patel V, Weiss H, et al. The incidence of psychoses in diverse settings, INTREPID (2): a feasibility study in India, Nigeria, and Trinidad. Psychological medicine. 2016;46(9):1923-33.
3. Cohen A, Padmavati R, Hibben M, Oyewusi S, John S, Esan O, et al. Concepts of madness in diverse settings: a qualitative study from the INTREPID project. BMC psychiatry. 2016;16(1):388.

For peer review only

Appendix 2: Ongoing and Planned Extensions

In addition to enabling us to investigate and test our primary research questions and hypotheses, INTREPID II establishes in each setting platforms and infrastructure for the conduct of other studies. Building on this, several extensions to INTREPID II are ongoing or planned. Here we highlight four.

(1) Data pooling for international comparisons

A particularly valuable aspect of INTREPID II is that our measures and methods are aligned with previous major research programmes on psychotic disorders in Europe to enable direct comparisons and data pooling to explore variation between countries and populations. For example, secondary hypotheses, regarding incidence rates, initial presentation, and risk factors will be tested by combining INTREPID II data with data from the UK, the Netherlands, France, Spain and Italy collected as part of the AESOP and EU-GEI studies (e.g., that, overall, incidence rates will be lower in India and Nigeria than in Trinidad and northern European countries).

(2) Genetics

Our current understanding of the genetics of psychotic disorders is limited by lack of diversity in the samples used. Most samples comprise individuals of European ancestry. Consequently, when applied to other populations, findings are less applicable. For example, polygenic risk scores (PRS), a measure of the total effects of multiple genes on risk (1), derived from large scale genome-wide association studies, explain far less of the variance in risk when applied in non-European samples (2). INTREPID II, then, provides an opportunity to generate samples from diverse populations that can contribute to global efforts to expand genetic studies to include people of African, Caribbean, and Asian ancestry.

Extending what we originally planned, participating cases and controls will now be invited to provide DNA samples at baseline via blood or saliva samples. Those who provide informed consent will have 10ml blood samples collected in EDTA tubes by a phlebotomy-trained researcher, or 2.5ml of saliva can be provided using Oragene saliva kits. Samples will be shipped to our partner organisations in the USA for DNA extraction and genome-wide association studies (GWAS), with extracted DNA samples then returned to the study settings. By pooling these data with larger datasets, our data will contribute to the generation of PRS that are applicable to our target populations, which we can then use to analyse genetic risk and gene-environment interactions within the INTREPID II cohort.

(3) Spatial Effects

The use of accessible technology to collect GPS coordinates for all participants, service providers and traditional healers provides an opportunity to explore spatial effects – in incidence, risk factors, outcomes, and help-seeking

For peer review only

– at the neighbourhood level. This will allow us to link individual data with ecological data in order to investigate risk and protective factors at the neighbourhood level, as well as facilitating geographical analyses of help-seeking behaviour.

(4) A Global Consortium

INTREPID II will act as a platform for the development of long-term international collaborations to address the lack of evidence on psychotic disorders in the Global South. INTREPID II provides a methodological template for epidemiological research on psychosis across diverse contexts, as exemplified by the establishment of a new research programme in South Africa that uses parallel methods, PSYMAP-ZN (led by Professor Bonginkosi Chiliza, University of KwaZulu-Natal, and Professor Jonathan Burns, University of Exeter). Building on these two programmes, we intend to establish a global consortium for population-based research involving INTREPID II and PSYMAP-ZN researchers and leading psychosis researchers from strategically-chosen settings in more diverse settings across the world – with a particular emphasis on areas where evidence is currently lacking – to extend this research agenda across geographic and disciplinary boundaries. The consortium will involve capacity building, data pooling, knowledge sharing platforms, the development of shared instruments and innovative methods, and will be underpinned by partnerships with service users and carers.

References

1. Consortium IS. Common polygenic variation contributes to risk of schizophrenia and bipolar disorder. Nature. 2009;460(7256):748.
2. Vilhjálmsson BJ, Yang J, Finucane HK, Gusev A, Lindström S, Ripke S, et al. Modeling linkage disequilibrium increases accuracy of polygenic risk scores. The American Journal of Human Genetics. 2015;97(4):576-92.

For peer review only

BMJ Open

INTREPID II: Protocol for a multi-study programme of research on untreated psychosis in India, Nigeria, and Trinidad

Journal:	*BMJ Open*
Manuscript ID	bmjopen-2020-039004.R1
Article Type:	Protocol
Date Submitted by the Author:	14-Apr-2020

Complete List of Authors:	Roberts, Tessa; Institute of Psychiatry Psychology and Neuroscience, Health Service & Population Research department; King's College London, ESRC Centre for Society and Mental Health<br>Gureje, Oye; University of Ibadan, WHO Collaborating Centre for Research and Training in Mental Health, Neuroscience and Substance Abuse, Department of Psychiatry<br>Thara, Rangaswamy; Schizophrenia Research Fondation<br>Hutchinson, Gerard; The University of the West Indies at Saint Augustine Faculty of Medical Sciences<br>Cohen, Alex; Harvard University T H Chan School of Public Health, Department of Epidemiology<br>Weiss, Helen; London School of Hygiene and Tropical Medicine, Epidemiology and Population Health; London School of Hygiene and Tropical Medicine<br>John, Sujit; Schizophrenia Research Foundation<br>Lee Pow, Joni; The University of the West Indies at Saint Augustine Faculty of Medical Sciences<br>Donald, Casswina; The University of the West Indies at Saint Augustine Faculty of Medical Sciences<br>Olley, Bola; University of Ibadan College of Medicine<br>Miguel Esponda, Georgina; Institute of Psychiatry Psychology and Neuroscience, Health Service and Population Research department; King's College London, ESRC Centre for Society and Mental Health<br>Murray, Robin; Institute of Psychiatry Psychology and Neuroscience, Department of Psychosis Studies<br>Morgan, Craig; Institute of Psychiatry Psychology and Neuroscience, Health Service and Population Research department; King's College London, ESRC Centre for Society and Mental Health

**Primary Subject Heading**:	Epidemiology
Secondary Subject Heading:	Mental health, Global health
Keywords:	Schizophrenia & psychotic disorders < PSYCHIATRY, EPIDEMIOLOGY, MENTAL HEALTH

SCHOLARONE™
Manuscripts

I, the Submitting Author has the right to grant and does grant on behalf of all authors of the Work (as defined in the below author licence), an exclusive licence and/or a non-exclusive licence for contributions from authors who are: i) UK Crown employees; ii) where BMJ has agreed a CC-BY licence shall apply, and/or iii) in accordance with the terms applicable for US Federal Government officers or employees acting as part of their official duties; on a worldwide, perpetual, irrevocable, royalty-free basis to BMJ Publishing Group Ltd ("BMJ") its licensees and where the relevant Journal is co-owned by BMJ to the co-owners of the Journal, to publish the Work in this journal and any other BMJ products and to exploit all rights, as set out in our licence.

The Submitting Author accepts and understands that any supply made under these terms is made by BMJ to the Submitting Author unless you are acting as an employee on behalf of your employer or a postgraduate student of an affiliated institution which is paying any applicable article publishing charge ("APC") for Open Access articles. Where the Submitting Author wishes to make the Work available on an Open Access basis (and intends to pay the relevant APC), the terms of reuse of such Open Access shall be governed by a Creative Commons licence – details of these licences and which Creative Commons licence will apply to this Work are set out in our licence referred to above.

Other than as permitted in any relevant BMJ Author's Self Archiving Policies, I confirm this Work has not been accepted for publication elsewhere, is not being considered for publication elsewhere and does not duplicate material already published. I confirm all authors consent to publication of this Work and authorise the granting of this licence.

For peer review only

INTREPID II: Protocol for a multi-study programme of research on untreated psychosis in India, Nigeria, and Trinidad

Tessa Roberts[1,8], Oye Gureje[2], Thara Rangaswamy[3], Gerard Hutchinson[4], Alex Cohen[5], Helen A. Weiss[6], Sujit John[3], Joni Lee Pow[4], Casswina Donald[4], Bola Olley[2], Georgina Miguel Esponda[1,8], Robin M. Murray[7], Craig Morgan*[1,8,9].

***Correspondence to**: Professor Craig Morgan, ESRC Centre for Society and Mental Health, Institute of Psychiatry, Psychology & Neuroscience, King's College London. Tel: 020 7848 0351. E-mail: craig.morgan@kcl.ac.uk

[1]Health Service and Population Research Department, Institute of Psychiatry, Psychology & Neuroscience, King's College London, UK; [2]WHO Collaborating Centre for Research and Training in Mental Health, Neuroscience and Substance Abuse, Department of Psychiatry, University of Ibadan, Ibadan, Nigeria; [3]Schizophrenia Research Foundation, Chennai, India; [4]Department of Psychiatry, University of the West Indies, Saint Augustine, Trinidad; [5]Department of Epidemiology, Harvard TH Chan School of Public Health, [6]MRC Tropical Epidemiology Group, Faculty of Epidemiology and Population Health, London School of Hygiene and Tropical Medicine, London, UK; [7]Psychosis Studies Department, Institute of Psychiatry, Psychology & Neuroscience, King's College London, London, UK; [8]ESRC Centre for Society and Mental Health, King's College London; [9]National Institute for Health Research (NIHR) Mental Health Biomedical Research Centre at South London and Maudsley NHS Foundation Trust and King's College London.

Funding statement

This programme is funded by the UK Medical Research Council (MRC) (MRC Reference: MR/PO25927/1). The authors acknowledge financial support from the National Institute for Health Research (NIHR) Specialist Biomedical Research Centre for Mental Health at South London and Maudsley NHS Foundation Trust and King's College London, and the ESRC Centre for Society and Mental Health at King's College London (ESRC Reference: ES/S012567/1). This programme builds on research funded by the Wellcome Trust (WT094525).

**Abstract Word Count**	250
**Text Body Word Count**	3866
**Tables**	2
**Figures**	2

For peer review only - http://bmjopen.bmj.com/site/about/guidelines.xhtml

Abstract

Introduction

There are few robust and directly comparable studies of the epidemiology of psychotic disorders in the Global South. INTREPID II is designed to investigate variations in untreated psychotic disorders in the Global South in (1) incidence and presentation, (2) 2-year course and outcome; (3) help-seeking and impact, and (4) physical health.

Methods

INTREPID II is a programme of research incorporating incidence, case-control, and cohort studies of psychoses in contiguous urban and rural areas in India, Nigeria, and Trinidad. In each country, the target samples are 240 untreated cases with a psychotic disorder, 240 age-, sex-, and neighbourhood-matched controls, and 240 relatives or caregivers. Participants will be followed, in the first instance, for 2 years. In each setting, we have developed and are employing comprehensive case-finding methods to ensure cohorts are representative of the target populations. Using methods developed during pilot work, extensive data are being collected at baseline and 2-year follow-up across several domains: clinical, social, help-seeking and impact, and biological.

Ethics and dissemination

Informed consent is sought, and participants are free to withdraw from the study at any time. Participants are referred to mental health services if not already in contact with these and emergency treatment arranged where necessary. All data collected is confidential, except when a participant presents a serious risk to either themselves or others. This programme has been approved by ethical review boards at all participating centres. Findings will be disseminated through international conferences, publications in international journals, and through local events for key stakeholders.

For peer review only

For peer review only - http://bmjopen.bmj.com/site/about/guidelines.xhtml

Article Summary

- Comprehensive case finding methods, building on extensive pilot work, to generate as complete a sample as possible and reduce selection bias,
- Inclusion of population-based, matched controls
- Direct comparability of methods across settings
- Potential trade-offs between cross-setting comparability and local validity
- Use of retrospective self-reports for several factors, which are potentially subject to recall bias and which create challenges in establishing the direction of associations

For peer review only

For peer review only - http://bmjopen.bmj.com/site/about/guidelines.xhtml

INTRODUCTION

Psychotic disorders, such as schizophrenia, affect more than 23 million people worldwide, contribute substantially to the global burden of disease, and are associated with high rates of disability and mortality (1-3). However, there are striking global inequities in our knowledge of psychoses. Over 85% of the world's population lives in Asia, Africa, Latin America, or the Caribbean (referred to here as the Global South)[1], but only a small fraction of research on psychotic disorders is done in these settings (4, 5). This has two implications. First, our knowledge of psychotic disorders, especially of the basic epidemiology, of associated risk factors, and of course and outcome, is incomplete and may be distorted. We do not know whether psychoses manifest, occur, and develop in the same ways around the world. Second, we do not have robust and replicated findings on which to base the development of accessible, humane, and effective services and public health initiatives in low resource settings. Conducting studies in a range of countries and contexts is essential to improve our understanding of the nature of psychotic disorders globally and to provide a much-needed evidence base to inform the development and implementation of effective interventions and services in diverse settings.

We established INTREPID II - the first multi-country study in four decades in the Global South – to extend our knowledge of psychotic disorders in diverse settings. This builds on extensive feasibility and pilot work (5-7) (INTREPID I; see Supplementary Materials, Appendix 1).

AIM, OBJECTIVES, RATIONALE

Our aim is to investigate variability in incidence, presentation, outcome, and impact of untreated psychotic disorders in three diverse countries of the Global South – India, Nigeria, and Trinidad – through four interconnected studies.

Study 1: Incidence, Presentation, and Risk

Objective: To investigate the incidence and presentation of untreated psychotic disorders in each setting and associated risk factors.

Psychotic disorders are highly heterogenous in incidence, presentation, and course and outcome. For example, the incidence of schizophrenia and other psychoses varies markedly across populations and social groups (8, 9). Rates are higher among men (9), in urban areas (10), and in many – but not all – migrant and minority ethnic populations (11). However, little is known about the incidence of psychoses in the Global South, beyond

[1] The term Global South refers to countries in Asia, Africa, Latin America and the Caribbean and does not necessarily refer to the geographical south, see e.g. http://www.fc-ssc.org/en/partnership_program/south_south_countries.

For peer review only - http://bmjopen.bmj.com/site/about/guidelines.xhtml

a small number of studies (e.g. (5, 12)), and we cannot assume that findings from the Global North generalise to other settings. There is tentative evidence, for example, that consistent findings from the Global North, such as the association with urbanicity, may not apply universally (13-15). Further, the phenomenology (i.e., symptom profile) of psychotic disorders is highly varied. Individuals experience a range of symptoms, in various combinations, spanning multiple dimensions, including symptoms of reality distortion (i.e., delusions, hallucinations), thought disturbance, mania, depression, and poverty of affect, speech, and volition. There is some evidence that symptom profiles vary across social and cultural contexts. For example, the Determinants of Outcome of Severe Mental Disorders (DOSMeD) study, a two year cohort study conducted in ten countries by the World Health Organization, found that non-affective acute remitting psychoses (i.e., presentations characterised by rapid onset, symptoms of reality distortion, and quick remission) were around ten times more common in settings in the Global South compared with the Global North (16), but these findings have not been replicated.

There is robust evidence from the Global North implicating an array of factors that likely combine in complex ways to increase risk. These include genetic (17, 18), neurodevelopmental markers (e.g., birth complications, poor premorbid function) (19, 20), exposure to trauma and other social disadvantages (21, 22), migration and minority ethnic status (11, 23), and substance use (24, 25). Further, there is growing evidence that specific risk factors are associated with particular symptoms (18, 26-28). For example, there is evidence of an association between social risk factors and specific symptoms of reality distortion (29-36), i.e. more delusions and hallucinations. It may be, then, that variations in incidence and presentation between settings reflect different population distributions in relevant risks. However, little research has explored these associations in the Global South.

Studying variations in incidence and presentation and associated risks in diverse populations may provide important insights into the aetiology of psychoses and provide a basis for developing public health strategies to reduce the burden of psychotic disorders. In this study, we will test several primary hypotheses on whether variations and associations observed in the Global North hold in more diverse settings.

Study 2: Course and Outcome

Objective: To investigate two-year course and outcome of psychotic disorders and associated factors.

The long-term course and outcome of psychoses following a first episode is highly variable. Evidence from the Global North suggests that, over a period of 5 to 10 years, around half of those with a psychotic disorder recover symptomatically (i.e., are symptom free for a period of 2 or more years) (37-40), but the proportion who achieve both symptom and social recovery is much lower (8-20%) (41), with high levels of enduring unemployment and social isolation (42-47). Several factors are associated with poor symptom and social outcomes, including premorbid difficulties, baseline symptom type (i.e., negative symptoms) and severity,

For peer review only - http://bmjopen.bmj.com/site/about/guidelines.xhtml

cognition, long duration of untreated psychosis, and persistent substance use (48-50). As with incidence and presentation, it seems that course and outcome vary by context. The DOSMeD study (51) and the International Study of Schizophrenia (ISoS) (12) reported better symptom and social outcomes for psychotic disorders in developing (i.e., Global South) vs. developed (i.e., Global North) countries, which has often been attributed to greater family support and community cohesion in more traditional societies. There are, however, several well documented methodological limitations to the DOSMeD and ISoS, not least that the number of countries included from the Global South is small (n, 3). Subsequent research appears to show greater variation between and within countries in the Global South (52).

In this study, we will describe and compare course and outcome at 2 years within and between settings and then test several primary hypotheses on the nature and origins of any observed variations.

Study 3: Help-seeking and Impact

Objective: To investigate: (a) help-seeking; and (b) the impact of psychotic disorders on individuals and families, using a combination of quantitative and qualitative approaches.

Many people with psychotic disorders in the Global South receive no formal (biomedical) treatment or begin treatment well after the critical window when early intervention is most effective (6, 53). Formal care in many countries often falls below minimum quality standards (54), and much of the burden of care falls on families. The use of traditional and religious healing for mental health problems is widespread in both Africa and Asia, even among those who also consult mental health services (55, 56). Such services also exist in the Caribbean, but are more disparate, less specialised, and typically used in addition – rather than as an alternative – to formal health services (5, 6). Practitioners of traditional medicine and faith healing fill a major gap in countries where formal care is scarce (57), but the nature and quality of the care they provide is highly variable (58). Human rights abuses have been widely documented in both traditional healing sites and formal mental health services around the world (59). In part because of this, family members provide a large proportion of care for people with long-standing problems – including severe mental disorders – in the Global South (60). Caring for a relative with a psychotic disorder can have a major physical, emotional and economic impact on families, particularly in households with limited resources (61-63). There is also evidence of high levels of stigma in many countries of the world, including India (64), Nigeria (65), and Trinidad (66).

To plan appropriate services and understand differences in outcomes, further in-depth evidence, both quantitative and qualitative, is needed about how individuals and families respond to psychotic disorders and their needs and experiences, including the treatment they receive, within local contexts. In this study, we will first describe and compare, between and within settings (e.g., by gender, by age, etc.), the types and extent of contacts with formal services and other providers and the impact (i.e., on quality of life) and burden of psychoses for individuals and families. We will then test, using quantitative data, several related primary

For peer review only - http://bmjopen.bmj.com/site/about/guidelines.xhtml

hypotheses and address, using in-depth qualitative data, questions concerning how individuals and families make sense of and respond to psychoses and the impacts on individuals and families.

Study 4: Physical Health

Objective: To investigate the types and prevalence of physical health problems and related biological markers.

In the Global North, those with a psychotic disorder have higher rates of physical health problems and higher rates of all-cause mortality (67), particularly cardiovascular disease and metabolic syndrome (68), which may result from both antipsychotic medication use and lifestyle factors (69-71). Comorbidity of physical and mental health problems is likely to impact negatively on quality of life and recovery (72). Our knowledge of the physical health of people with psychoses in the Global South is much more limited (73), but suggests that there is also a mortality gap compared with the general population and this may be related to similar health problems as in the Global North (74, 75). For example, evidence from India suggests that metabolic syndrome is common (76) and there are rising rates of diabetes and cardiovascular disease in India (77), Nigeria (78), and Trinidad (79). It may also be, however, that the types of physical health problems (e.g., malnutrition; infectious diseases; injury due to accident, violence) in developing countries differ from those common in developed countries.

In this study, we will describe and compare, between and within settings, markers and measures of physical health problems between cases and age- and sex-matched controls, and test hypotheses concerning the nature and origins of variations in physical health within and between settings.

FEASIBILITY AND PILOT WORK

See Appendix 1 in our Supplementary Materials for a description of our feasibility and pilot work.

SETTINGS

INTREPID II is a collaboration between the Schizophrenia Research Foundation (SCARF; Chennai), the University of Ibadan (Nigeria), the University of the West Indies at St Augustine (Trinidad), the London School of Hygiene and Tropical Medicine (UK), and the Institute of Psychiatry, Psychology & Neuroscience, King's College London (UK).

The study settings, in India, Nigeria, and Trinidad, were selected to maximise potential comparisons between sites and with existing datasets. They represent three economically, socially, and culturally diverse areas, on three continents, each undergoing rapid economic and social transformations.

For peer review only - http://bmjopen.bmj.com/site/about/guidelines.xhtml

For peer review only

In each setting, our catchment areas comprise urban and rural areas with total populations of around 500,000 adults aged 18-64 years. In Nigeria, the catchment area comprises three contiguous Local Government Areas in and around the city of Ibadan in Oyo State: Ibadan North East, Ibadan South East, and Ona-Ara (total adult population ~584,000, population density 914 – 18,356 per km^2). In Trinidad, the catchment area comprises the municipalities of Arima, Tunapuna-Piarco, Chaguanas, Port of Spain, San Juan/Laventille, Diego Martin, and Sangre Grande (total adult population ~487,000, population density 82 – 3,090 per km^2). In India the catchment area consists of three contiguous taluks, Chengelpettu, Uthiramerur, and Maduranthakam, located south of Chennai, in the district of Kancheepuram in the state of Tamil Nadu (total adult population ~600,000, population density 361 – 737 per km^2).

METHODS

Overview

INTREPID II comprises four interconnected studies (Figure 1; See Strobe Statement, Supplementary Materials). As a basis for these studies, we are identifying, assessing, and following, in each catchment area, population-based cohorts of cases (individuals with an untreated psychotic disorder) and controls (individuals with no history of a psychotic disorder) (Figure 2).

[Insert Figures 1 and 2]

In each setting, using methods and infrastructure developed during our feasibility and pilot work, INTREPID I, we will identify, assess, and follow at 2 years cohorts of 240 untreated (incident) cases with a psychotic disorder (total, 720) and 240 matched controls (total, 720), using methods developed in INTREPID I. Our inclusion and exclusion criteria for cases are in line with those used in previous studies, including the WHO multi-country studies (12), and are purposefully broad to capture heterogeneity and to allow sub-analyses by duration of untreated psychosis (Table 1).

Table 1. Inclusion and exclusion criteria.

	Inclusion criteria	Exclusion criteria
Cases	• Age 18 to 64 years<br>• Currently resident in catchment area (primary residence)<br>• Presence of ICD-10 psychotic disorder, including substance-induced psychoses<br>• Not treated with antipsychotic medication for more than one	• Transient psychotic symptoms resulting from acute intoxication as defined by ICD-10<br>• Moderate or severe learning disability, as defined by ICD-10<br>• Clinically manifest organic cerebral disorder (e.g. infections, parasitic, toxic, cerebrovascular, epilepsy, brain injury), as

For peer review only

For peer review only - http://bmjopen.bmj.com/site/about/guidelines.xhtml

	continuous month prior to the start of initial case identification	defined by ICD-10
Controls	• Age 18 to 64 years<br>• Currently resident in catchment area (primary residence)<br>• Same gender as index case<br>• Within 5 years of age of index case	• Past or current ICD-10 psychotic disorder<br>• Moderate or severe learning disability, as defined by ICD-10<br>• Clinically manifest organic cerebral disorder (e.g. infections, parasitic, toxic, cerebrovascular, epilepsy, brain injury), as defined by ICD-10
Relatives	• Age 18 and above<br>• Relative or carer of a case who has consented to participate in the current study	• Insufficient contact with case to provide information on family burden or mental health

Sample (1) Cases

To estimate incidence, we aim to identify all individuals with an untreated psychotic disorder (cases) within each catchment area. Untreated is defined as never having received treatment with anti-psychotic medication for one continuous month prior to the start of the case-finding period.

In each catchment area, we are using a multi-pronged approach to case identification. First, using procedures developed in INTREPID I, we have established comprehensive case detection systems by mapping and seeking to engage a comprehensive set of service providers and community key informants who may encounter individuals with psychotic disorders within the catchment area. This includes the professional sector (specialist and generalist services; public, private and third sector), the folk sector (including traditional and religious services), and the popular sector (i.e. informal sources of support). Second, we give providers and informants materials developed in our pilot work that detail, using local terms and language, the experiences and behaviours that characterise psychosis. Third, in each catchment area, researchers check with each provider and informant regularly and conduct regular checks of admissions ledgers and registers for in-patient and out-patient services (where these exist), to identify potential cases. In addition, in rural villages in Chennai and Ibadan, field workers visit village meeting points to enquire about potential cases. Potential cases are then screened for inclusion using the Screening Schedule for Psychosis (51), an instrument that has been widely used in epidemiological studies of psychoses. Those who screen positive and who meet inclusion criteria are approached and informed consent sought.

For peer review only - http://bmjopen.bmj.com/site/about/guidelines.xhtml

Case-finding began on 1 May 2018 and will conclude 30 April 2020. At the end of the case-finding period, we will conduct leakage studies in each setting to identify possible cases meeting our inclusion criteria who may not have been identified. Each research team will systematically re-check admissions ledgers and registers for in-patient and out-patient services and complete final checks with healers and key informants.

All eligible cases identified through the incidence study are invited to participate in the programme. Rates of refusal are documented and basic data (i.e. age, gender, area of residence, sector of identification, and where possible ethnicity, religion, duration of untreated psychosis and mode of onset) is collected for those who decline to participate, or who it is not possible to interview, to assess non-response bias.

Sample (2) Controls

Age-, sex- and neighbourhood-matched controls are recruited to provide indicative population data against which to compare cases in terms of hypothesised risk factors, social outcomes, and physical health. We use the Psychosis Screening Questionnaire to collect information on any current or past experiences of psychosis (80). In the absence of a readily accessible sampling frame to randomly select potential controls, we map the ten nearest neighbouring households for each case, listing all residents in these dwellings by sex and age. All potential controls for the case (defined as the same gender and ±5 years of age) are then approached in random order, until an eligible control is identified. When no match is identified the process is repeated. This approach was successfully piloted in all settings.

Sample (3) Relatives and Caregivers

We seek consent from each case to approach a close relative or caregiver to participate in the study. We then approach each designated relative to seek his/her consent. The primary purposes of including relatives are to corroborate and extend information from cases (e.g., physical health and illness), to collect information on premorbid adjustment, family history of mental disorder, and other risk factors, and to collect information on family responses to psychosis, help-seeking, and impact (burden) on family.

Follow-up

All participants will be followed at 2 years. To facilitate this, we collect detailed contact information at baseline (address, telephone number, email address if applicable, service provider details) from each case and control, including details of a relative or friend who can be contacted to trace the individual. In addition, to maintain contact and minimise attrition, we contact participants every six months, by telephone or in person, to confirm or update contact details. Based on our pilot work, we expect to re-assess around 80% of cases and controls 2 years after initial identification.

For peer review only - http://bmjopen.bmj.com/site/about/guidelines.xhtml

Sample size

In each setting, we anticipate (based on pilot findings) identifying around 300 untreated incident cases. Of those, given an expected refusal rate of 20% of all eligible cases (based on our pilot work), we anticipate recruiting approximately 240 cases (total, 720), and 240 individually matched controls (total, 720). These sample sizes are larger than most previous studies (5, 52) and provide good statistical power to test our hypotheses (i.e., > 80% at p 0.05). For example: (a) with samples of around 300 untreated incident cases in each setting, we will have over 80% power to detect an incidence rate ratio of 1.5 (or greater) between two areas (e.g., urban vs. rural), if the incidence rate in the lowest risk area is 20 per 100,000; (b) with a sample of 240 cases and 240 controls in each setting, we will have over 80% power to detect an odds ratio of 2.0 (or greater) in case-control comparisons when the prevalence of exposure (risk factor) is at least 15% in controls; (c) using gender as an example, with a sample of 192 cases followed at 2 years in each setting, we will have 80% power (or greater) to detect a difference in the proportion of cases with a poor outcome (e.g., continuously psychotic) of 0.20 (20%) or greater, when the proportion of men with a poor outcome is 0.40 and the proportion of women is 0.20 (i.e., equivalent to an odds ratio of ~ 2.5).

Data collection

To test the hypotheses and address the research questions of our 4 studies, we collect information from cases, relatives, and controls at baseline and at 2 year follow up. A summary of the measures and the study to which they relate is provided in Table 2. All, where necessary, have been translated into local languages and back translated to check equivalence.

All those who consent are interviewed and assessed by trained research workers using structured instruments and protocols either at home or at a local clinic. For participants who are in contact with health services, interview data are supplemented with reference to clinical notes, with participants' consent.

Interviews and assessments are conducted by researchers fluent in the local language. To ensure consistency of methods across settings, all researchers are fully trained using a mixture of online materials and exercises, with feedback, and face to face training, delivered both by the UK team and locally by senior researchers under the supervision of the country principal investigators (PIs). All PIs are experienced psychiatrists with extensive backgrounds in both national and international research. Inter-rater reliability for core instruments that require rater judgement will be tested regularly across settings using video-recorded interviews with cases and controls to ensure that the measures are applied consistently throughout the duration of the programme. Responses will be triangulated with relative reports and, where applicable, clinical records.

For peer review only - http://bmjopen.bmj.com/site/about/guidelines.xhtml

Table 2. Timing and participants for each measure used in the INTREPID II programme.

	Study	Baseline			2-year follow-up		
		Untreated cases (n, 720)	Relatives (n, 720)	Controls (n, 720)	Untreated cases (n, ~ 576)	Relatives (n, ~ 576)	Controls (n, ~ 576)
MRC Sociodemographic Schedule*	1,2,3,4	✓	✓	✓	✓	✓	✓
Personal and Psychiatric History Schedule (PPHS): Baseline* (16)	1,3	✓	✓	-	-	-	-
Personal and Psychiatric History Schedule (PPHS): Follow-up* (16)	2,3	-	-	-	✓	✓	-
WHO Life Chart* (84)	2,3	-	-	-	✓	✓	-
Schedules for Clinical Assessment in Neuropsychiatry (SCAN)* (85)	1,2	✓	-	-	✓	-	-
General Assessment of Functioning (GAF) - Symptoms & Disability scales* (86)	1,2	✓	-	✓	✓	-	✓
WHO Disability Assessment Schedule (DAS)* (87)	1,2	✓	✓	✓	✓	✓	✓
PANSS* (88)	1,2	✓	-	-	✓	-	-
Brief Assessment of Cognition in Schizophrenia (BACS) (89)	1,2	✓	-	✓	✓	-	✓
Family Interview for Genetic Studies (FIGS) (90)	1,2	✓	✓	✓	-	-	-
Premorbid Adjustment Scale (PAS) (91)	1,2	✓	✓	-	-	-	-
Alcohol, Smoking and Substance Involvement Screening Test (ASSIST) (92)	1,2	✓	-	✓	✓	-	✓
Childhood Trauma Questionnaire (CTQ) (93)	1,2	✓	-	✓	-	-	-
Harvard Trauma Questionnaire (HTQ) (94)	1,2	✓	-	✓	✓	-	✓
List of Threatening Events (LoTE) (95)	1,2	✓	-	✓	✓	-	✓

For peer review only

CIDI support networks module	1,2	✓	-	✓	✓	-	✓
Family Burden Interview Schedule (FBIS) (96, 97)	3	-	✓	-	-	✓	-
McGill Illness Narrative Interview (MINI) (98)	3	✓	✓	-	✓	✓	-
WHO STEPS (99)	4	✓	-	✓	✓	-	✓
Blood pressure	4	✓	-	✓	✓	-	✓
Blood tests	4	✓	-	✓	✓	-	✓
Screen for TB	4	✓	-	✓	✓	-	✓
Medication checklist	4	✓	-	✓	✓	-	✓
Glasgow Antipsychotic Side-effect Scale (100)	4	✓	-	-	✓	-	-
Blood sample for genetics	1	✓	-	✓	-	-	-
GPS coordinates	1,3	✓	-	✓	-	-	-

* Indicates core instruments

For peer review only

Reliability

All measures will be applied identically, by the same research team, for both cases and controls (where measures apply to both groups). Researchers from across the field settings rated video-taped interviews at study onset and their ratings were compared to gold standard responses developed by the PIs. The mean and range for the proportion of scores that matched the gold standard ratings for each instrument, or were within an acceptable margin, were as follows: Schedules for Clinical Assessment in Neuropsychiatry (SCAN), 87% (85%-88%); Disability Assessment Schedule (DAS) 88% (85%-92%); Personal and Psychiatric History Schedule (PPHS) 76% (73%-84%); Global Assessment of Function 12.5% (0%-50%). Feedback was provided to the research workers and their ratings will continue to be monitored at repeated intervals throughout the study.

Analysis Plan

We will use standard summary statistics, with indicators of spread and precision as appropriate (e.g., crude incidence rates per 100,000 person years, with 95% confidence intervals) to describe the data. We will then use appropriate regression models to compare data between and within settings (e.g., Poisson regression for incidence rates and other count data; Cox regression for time-to-event data; logistic regression (including multinomial) for categorical data [e.g., course type]; and linear regression for continuous data [e.g., GAF score, blood pressure]). In building regression models, we will first fit univariable models, then test for effect modification by core variables (e.g., gender, age, setting, and time), and finally adjust for putative confounders of each hypothesised association by fitting multivariable models.

Where appropriate, we will use multiple imputation to deal with missing data. In addition, or where assumptions necessary for imputation are not met, we will (re) conduct analyses on participants with complete data only. Where possible, analyses based on imputed data will be presented, with complete data analyses presented as sensitivity analyses in supplementary materials.

Framework Analysis will be used to analyse qualitative data (81), adopting an iterative process of reading and annotating transcripts to identify salient themes, which will form the basis for comparisons between and within settings.

ETHICS AND DISSEMINATION

Informed consent will be sought from all eligible participants, and participants will be free to withdraw from the study at any time. Capacity to consent will be assessed by trained researchers at the point of seeking consent. If at any point, there is concern for the mental or physical health or welfare of participants, researchers will discuss immediately with the country PI, who will arrange for assessment and referral to the appropriate local mental or other health service, including emergency treatment where necessary.

For peer review only

All data collected will be kept confidential, except with the express consent of the patient to share information with health care professionals, or in cases where the participant poses a serious risk either to themselves or to others.

This study has been approved by the ethical review boards of King's College London (reference number: HR-17/18-5601), London, UK; London School of Hygiene and Tropical Medicine (reference number: 15807), the Schizophrenia Research Foundation, Chennai, India; the University of Ibadan, Ibadan, Nigeria; the University of the West Indies, St Augustine, Trinidad; and the North West, North Central, and Eastern Regional Health Authorities of Trinidad.

We will disseminate our findings widely, including through international conferences and publications in international journals, and through locally organised events for service users, service providers, and policy makers.

PATIENT AND PUBLIC INVOLVEMENT

Patients and members of the public were not involved in the design or conduct of the study. However, the research teams in each study setting are liaising with local service user and family organisations to discuss the interpretation of the findings, to consider potential recommendations arising from the evidence generated, and to devise and implement local dissemination plans.

ONGOING AND PLANNED EXTENSIONS

In addition to enabling us to investigate and test our primary research questions and hypotheses, INTREPID II establishes in each setting platforms and infrastructure for the conduct of other studies. Building on this, several extensions to INTREPID II are ongoing or planned. Four of these are detailed in Appendix 2 (see Supplementary Materials, Appendix 2).

For peer review only

For peer review only - http://bmjopen.bmj.com/site/about/guidelines.xhtml

REFERENCES

1. Saha S, Chant D, McGrath J. A systematic review of mortality in schizophrenia: is the differential mortality gap worsening over time? Archives of General Psychiatry. 2007;64(10):1123-31.
2. McGrath J, Saha S, Chant D, Welham J. Schizophrenia: a concise overview of incidence, prevalence, and mortality. Epidemiologic Reviews. 2008;30(1):67-76.
3. Charlson FJ, Ferrari AJ, Santomauro DF, Diminic S, Stockings E, Scott JG, et al. Global Epidemiology and Burden of Schizophrenia: Findings From the Global Burden of Disease Study 2016. Schizophrenia Bulletin. 2018.
4. McGrath J, Saha S, Welham J, El Saadi O, MacCauley C, Chant D. A systematic review of the incidence of schizophrenia: the distribution of rates and the influence of sex, urbanicity, migrant status and methodology. BMC Medicine. 2004;2(1):13.
5. Morgan C, John S, Esan O, Hibben M, Patel V, Weiss H, et al. The incidence of psychoses in diverse settings, INTREPID (2): a feasibility study in India, Nigeria, and Trinidad. Psychological Medicine. 2016;46(9):1923-33.
6. Morgan C, Hibben M, Esan O, John S, Patel V, Weiss HA, et al. Searching for psychosis: INTREPID (1): systems for detecting untreated and first-episode cases of psychosis in diverse settings. Social Psychiatry and Psychiatric Epidemiology. 2015;50(6):879-93.
7. Cohen A, Padmavati R, Hibben M, Oyewusi S, John S, Esan O, et al. Concepts of madness in diverse settings: a qualitative study from the INTREPID project. BMC Psychiatry. 2016;16(1):388.
8. Jongsma HE, Turner C, Kirkbride JB, Jones PB. International incidence of psychotic disorders, 2002–17: a systematic review and meta-analysis. The Lancet Public Health. 2019;4(5):e229-e44.
9. McGrath JJ. The surprisingly rich contours of schizophrenia epidemiology. Archives of General Psychiatry. 2007;64(1):14-6.
10. March D, Hatch SL, Morgan C, Kirkbride JB, Bresnahan M, Fearon P, et al. Psychosis and place. Epidemiologic Reviews. 2008;30(1):84-100.
11. Morgan C, Charalambides M, Hutchinson G, Murray RM. Migration, ethnicity, and psychosis: toward a sociodevelopmental model. Schizophrenia Bulletin. 2010;36(4):655-64.
12. Hopper K, Harrison G, Janca A, Norman Sartorius M. Recovery from schizophrenia: An international perspective: A report from the WHO Collaborative Project, the international study of schizophrenia: Oxford University Press; 2007.
13. Menezes PR, Scazufca M, Busatto GF, Coutinho LM, McGUIRE PK, Murray RM. Incidence of first-contact psychosis in Sao Paulo, Brazil. The British Journal of Psychiatry. 2007;191(51):s102-s6.
14. Jongsma HE, Gayer-Anderson C, Lasalvia A, Quattrone D, Mulè A, Szöke A, et al. Treated incidence of psychotic disorders in the multinational EU-GEI study. JAMA Psychiatry. 2018;75(1):36-46.
15. DeVylder JE, Kelleher I, Lalane M, Oh H, Link BG, Koyanagi A. Association of urbanicity with psychosis in low-and middle-income countries. JAMA Psychiatry. 2018;75(7):678-86.

For peer review only - http://bmjopen.bmj.com/site/about/guidelines.xhtml

16. Susser E, Wanderling J. Epidemiology of nonaffective acute remitting psychosis vs schizophrenia. Arch Gen Psychiatry. 1994;51:294-301.
17. Van Os J, Marcelis M, Sham P, Jones P, Gilvarry K, Murray R. Psychopathological syndromes and familial morbid risk of psychosis. The British Journal of Psychiatry. 1997;170(3):241-6.
18. Peralta V, Cuesta MJ. The relationship between syndromes of the psychotic illness and familial liability to schizophrenia and major mood disorders. Schizophrenia Research. 2007;91(1-3):200-9.
19. Tarbox SI, Addington J, Cadenhead KS, Cannon TD, Cornblatt BA, Perkins DO, et al. Premorbid functional development and conversion to psychosis in clinical high-risk youths. Development and Psychopathology. 2013;25(4pt1):1171-86.
20. Lyngberg K, Buchy L, Liu L, Perkins D, Woods S, Addington J. Patterns of premorbid functioning in individuals at clinical high risk of psychosis. Schizophrenia Research. 2015;169(1-3):209-13.
21. Morgan C, Gayer-Anderson C. Childhood adversities and psychosis: evidence, challenges, implications. World Psychiatry. 2016;15(2):93-102.
22. Read J, van Os J, Morrison AP, Ross CA. Childhood trauma, psychosis and schizophrenia: a literature review with theoretical and clinical implications. Acta Psychiatrica Scandinavica. 2005;112(5):330-50.
23. Selten J-P, van der Ven E, Termorshuizen F. Migration and psychosis: a meta-analysis of incidence studies. Psychological Medicine. 2019:1-11.
24. Di Forti M, Marconi A, Carra E, Fraietta S, Trotta A, Bonomo M, et al. Proportion of patients in south London with first-episode psychosis attributable to use of high potency cannabis: a case-control study. The Lancet Psychiatry. 2015;2(3):233-8.
25. Marconi A, Di Forti M, Lewis CM, Murray RM, Vassos E. Meta-analysis of the association between the level of cannabis use and risk of psychosis. Schizophrenia Bulletin. 2016;42(5):1262-9.
26. van Nierop M, Viechtbauer W, Gunther N, Van Zelst C, De Graaf R, Ten Have M, et al. Childhood trauma is associated with a specific admixture of affective, anxiety, and psychosis symptoms cutting across traditional diagnostic boundaries. Psychological Medicine. 2015;45(6):1277-88.
27. Van Os J, Jones P, Sham P, Bebbington P, Murray R. Risk factors for onset and persistence of psychosis. Social Psychiatry and Psychiatric Epidemiology. 1998;33(12):596-605.
28. Allardyce J, McCreadie RG, Morrison G, van Os J. Do symptom dimensions or categorical diagnoses best discriminate between known risk factors for psychosis? SocialPsychiatry and Psychiatric Epidemiology. 2007;42(6):429-37.
29. Demjaha A, Morgan K, Morgan C, Dazzan P, Landau S, Dean K, et al., editors. Symptom dimensions and ethnicity in the AESOP first onset psychosis study. Schizophrenia Research; 2006: ELSEVIER SCIENCE BV PO BOX 211, 1000 AE AMSTERDAM, NETHERLANDS.
30. Berg AO, Andreassen OA, Aminoff SR, Romm KL, Hauff E, Melle I. The impact of immigration and visible minority status on psychosis symptom profile. Social Psychiatry and Psychiatric Epidemiology. 2014;49(11):1747-57.

For peer review only - http://bmjopen.bmj.com/site/about/guidelines.xhtml

31. van der Ven E, Bourque F, Joober R, Selten J-P, Malla AK. Comparing the clinical presentation of first-episode psychosis across different migrant and ethnic minority groups in Montreal, Quebec. The Canadian Journal of Psychiatry. 2012;57(5):300-8.
32. Oher F, Demjaha A, Jackson D, Morgan C, Dazzan P, Morgan K, et al. The effect of the environment on symptom dimensions in the first episode of psychosis: a multilevel study. Psychological Medicine. 2014;44(11):2419-30.
33. Demjaha A, MacCabe JH, Murray RM. How genes and environmental factors determine the different neurodevelopmental trajectories of schizophrenia and bipolar disorder. Schizophrenia Bulletin. 2011;38(2):209-14.
34. Demjaha A, Morgan K, Morgan C, Landau S, Dean K, Reichenberg A, et al. Combining dimensional and categorical representation of psychosis: the way forward for DSM-V and ICD-11? Psychological Medicine. 2009;39(12):1943-55.
35. Bentall RP, de Sousa P, Varese F, Wickham S, Sitko K, Haarmans M, et al. From adversity to psychosis: pathways and mechanisms from specific adversities to specific symptoms. Social psychiatry and Psychiatric Epidemiology. 2014;49(7):1011-22.
36. Quattrone D, Di Forti M, Gayer-Anderson C, Ferraro L, Jongsma HE, Tripoli G, et al. Transdiagnostic dimensions of psychopathology at first episode psychosis: findings from the multinational EU-GEI study. Psychological Medicine. 2019;49(8):1378-91.
37. Morgan C, Lappin J, Heslin M, Donoghue K, Lomas B, Reininghaus U, et al. Reappraising the long-term course and outcome of psychotic disorders: the AESOP-10 study. Psychological Medicine. 2014;44(13):2713-26.
38. Jobe TH, Harrow M. Long-term outcome of patients with schizophrenia: a review. The Canadian Journal of Psychiatry. 2005;50(14):892-900.
39. Harrow M, Grossman LS, Jobe TH, Herbener ES. Do patients with schizophrenia ever show periods of recovery? A 15-year multi-follow-up study. Schizophrenia Bulletin. 2005;31(3):723-34.
40. Harrison G, Hopper K, Craig T, Laska E, Siegel C, Wanderling J, et al. Recovery from psychotic illness: a 15-and 25-year international follow-up study. The British Journal of Psychiatry. 2001;178(6):506-17.
41. Jääskeläinen E, Juola P, Hirvonen N, McGrath JJ, Saha S, Isohanni M, et al. A systematic review and meta-analysis of recovery in schizophrenia. Schizophrenia Bulletin. 2012;39(6):1296-306.
42. Stain HJ, Galletly CA, Clark S, Wilson J, Killen EA, Anthes L, et al. Understanding the social costs of psychosis: the experience of adults affected by psychosis identified within the second Australian National Survey of Psychosis. Australian & New Zealand Journal of Psychiatry. 2012;46(9):879-89.
43. Killaspy H, White S, Lalvani N, Berg R, Thachil A, Kallumpuram S, et al. The impact of psychosis on social inclusion and associated factors. International Journal of Social Psychiatry. 2014;60(2):148-54.
44. Gayer-Anderson C, Morgan C. Social networks, support and early psychosis: a systematic review. Epidemiology and Psychiatric Sciences. 2013;22(2):131-46.

For peer review only - http://bmjopen.bmj.com/site/about/guidelines.xhtml

45. Waghorn G, Saha S, Harvey C, Morgan VA, Waterreus A, Bush R, et al. 'Earning and learning'in those with psychotic disorders: The second Australian national survey of psychosis. Australian & New Zealand Journal of Psychiatry. 2012;46(8):774-85.

46. Kooyman I, Dean K, Harvey S, Walsh E. Outcomes of public concern in schizophrenia. The British Journal of Psychiatry. 2007;191(S50):s29-s36.

47. Marwaha S, Johnson S, Bebbington P, Stafford M, Angermeyer MC, Brugha T, et al. Rates and correlates of employment in people with schizophrenia in the UK, France and Germany. The British Journal of Psychiatry. 2007;191(1):30-7.

48. Menezes N, Arenovich T, Zipursky R. A systematic review of longitudinal outcome studies of first-episode psychosis. Psychological Medicine. 2006;36(10):1349-62.

49. Alvarez-Jimenez M, Priede A, Hetrick S, Bendall S, Killackey E, Parker A, et al. Risk factors for relapse following treatment for first episode psychosis: a systematic review and meta-analysis of longitudinal studies. SchizophreniaResearch. 2012;139(1-3):116-28.

50. Díaz-Caneja CM, Pina-Camacho L, Rodríguez-Quiroga A, Fraguas D, Parellada M, Arango C. Predictors of outcome in early-onset psychosis: a systematic review. npj Schizophrenia. 2015;1:14005.

51. Jablensky A, Sartorius N, Ernberg G, Anker M, Korten A, Cooper JE, et al. Schizophrenia: manifestations, incidence and course in different cultures A World Health Organization Ten-Country Study. Psychological Medicine Monograph Supplement. 1992;20:1-97.

52. Cohen A, Patel V, Thara R, Gureje O. Questioning an axiom: better prognosis for schizophrenia in the developing world? Schizophrenia Bulletin. 2007;34(2):229-44.

53. Demyttenaere K, Bruffaerts R, Posada-Villa J, Gasquet I, Kovess V, Lepine J, et al. Prevalence, severity, and unmet need for treatment of mental disorders in the World Health Organization World Mental Health Surveys. JAMA. 2004;291(21):2581-90.

54. Asher L, Fekadu A, Hanlon C. Global mental health and schizophrenia. Current Opinion in Psychiatry. 2018;31(3):193-9.

55. Burns JK, Tomita A. Traditional and religious healers in the pathway to care for people with mental disorders in Africa: a systematic review and meta-analysis. Social Psychiatry and Psychiatric Epidemiology. 2015;50(6):867-77.

56. Thirthalli J, Zhou L, Kumar K, Gao J, Vaid H, Liu H, et al. Traditional, complementary, and alternative medicine approaches to mental health care and psychological wellbeing in India and China. The Lancet Psychiatry. 2016;3(7):660-72.

57. Gureje O, Nortje G, Makanjuola V, Oladeji BD, Seedat S, Jenkins R. The role of global traditional and complementary systems of medicine in the treatment of mental health disorders. The Lancet Psychiatry. 2015;2(2):168-77.

58. Nortje G, Oladeji B, Gureje O, Seedat S. Effectiveness of traditional healers in treating mental disorders: a systematic review. The Lancet Psychiatry. 2016;3(2):154-70.

For peer review only - http://bmjopen.bmj.com/site/about/guidelines.xhtml

59. Drew N, Funk M, Tang S, Lamichhane J, Chávez E, Katontoka S, et al. Human rights violations of people with mental and psychosocial disabilities: an unresolved global crisis. The Lancet. 2011;378(9803):1664-75.

60. Thrush A, Hyder A. The neglected burden of caregiving in low-and middle-income countries. Disability and Health Journal. 2014;7(3):262-72.

61. Awad AG, Voruganti LN. The burden of schizophrenia on caregivers. Pharmacoeconomics. 2008;26(2):149-62.

62. Caqueo-Urízar A, Gutiérrez-Maldonado J, Miranda-Castillo C. Quality of life in caregivers of patients with schizophrenia: a literature review. Health and Quality of Life Outcomes. 2009;7(1):84.

63. Chan SW-c. Global perspective of burden of family caregivers for persons with schizophrenia. Archives of Psychiatric Nursing. 2011;25(5):339-49.

64. Loganathan S, Murthy SR. Experiences of stigma and discrimination endured by people suffering from schizophrenia. Indian Journal of Psychiatry. 2008;50(1):39.

65. Gureje O, Lasebikan VO, Ephraim-Oluwanuga O, Olley BO, Kola L. Community study of knowledge of and attitude to mental illness in Nigeria. The British Journal of Psychiatry. 2005;186(5):436-41.

66. Hutchinson G, Neehall JE, Simeon DT, Littlewood R. Perceptions about mental illness among pre-clinical medical students in Trinidad & Tobago. The West Indian Medical Journal. 1999;48(2):81-4.

67. Reininghaus U, Dutta R, Dazzan P, Doody GA, Fearon P, Lappin J, et al. Mortality in schizophrenia and other psychoses: a 10-year follow-up of the ÆSOP first-episode cohort. Schizophrenia Bulletin. 2014;41(3):664-73.

68. Doherty AM, Gaughran F. The interface of physical and mental health. Social psychiatry and psychiatric epidemiology. 2014;49(5):673-82.

69. Meyer JM, Stahl SM. The metabolic syndrome and schizophrenia. Acta Psychiatrica Scandinavica. 2009;119(1):4-14.

70. HERT M, Schreurs V, Vancampfort D, Winkel R. Metabolic syndrome in people with schizophrenia: a review. World Psychiatry. 2009;8(1):15-22.

71. Mitchell AJ, Vancampfort D, De Herdt A, Yu W, De Hert M. Is the prevalence of metabolic syndrome and metabolic abnormalities increased in early schizophrenia? A comparative meta-analysis of first episode, untreated and treated patients. Schizophrenia Bulletin. 2012;39(2):295-305.

72. Firth J, Siddiqi N, Koyanagi A, Siskind D, Rosenbaum S, Galletly C, et al. The Lancet Psychiatry Commission: a blueprint for protecting physical health in people with mental illness. The Lancet Psychiatry. 2019;6(8):675-712.

73. Hjorthøj C, Stürup AE, McGrath JJ, Nordentoft M. Years of potential life lost and life expectancy in schizophrenia: a systematic review and meta-analysis. The Lancet Psychiatry. 2017;4(4):295-301.

74. Fekadu A, Medhin G, Kebede D, Alem A, Cleare AJ, Prince M, et al. Excess mortality in severe mental illness: 10-year population-based cohort study in rural Ethiopia. The British Journal of Psychiatry. 2015;206(4):289-96.

For peer review only - http://bmjopen.bmj.com/site/about/guidelines.xhtml

75. Lêng CH, Chou MH, Lin S-H, Yang YK, Wang J-D. Estimation of life expectancy, loss-of-life expectancy, and lifetime healthcare expenditures for schizophrenia in Taiwan. Schizophrenia Research. 2016;171(1-3):97-102.

76. Padmavati R. Metabolic syndrome, serious mental illnesses & lifestyle. The Indian Journal of Medical Research. 2016;143(4):395.

77. Mohan V, Sandeep S, Deepa R, Shah B, Varghese C. Epidemiology of type 2 diabetes: Indian scenario. Indian Journal of Medical Research. 2007;125(3):217.

78. Azevedo M, Alla S. Diabetes in sub-saharan Africa: kenya, mali, mozambique, Nigeria, South Africa and zambia. International Journal of Diabetes in Developing Countries. 2008;28(4):101.

79. Joseph L. An assessment of knowledge, attitude, beliefs and risk perception of type 2 diabetes mellitus among the adult population of Trinidad and Tobago. 2016.

80. Bebbington P, Nayani T. The psychosis screening questionnaire. International Journal of Methods in Psychiatric Research. 1995.

81. Gale NK, Heath G, Cameron E, Rashid S, Redwood S. Using the framework method for the analysis of qualitative data in multi-disciplinary health research. BMC Medical Research Methodology. 2013;13(1):117.

For peer review only

Author Contributions

TR contributes to the overall coordination of the programme and drafted and revised the manuscript, based on the funded grant proposal written by CM, OG, RT, GH, HW, AC, and RM to the UK Medical Research Council.

RT, OG, GH contributed to the design the programme, lead the programme in India, Nigeria, and Trinidad, respectively, and contributed to review and revision of the manuscript.

AC, HW, RM contributed to the design of the programme and to review and revision of the manuscript.

GME contributes to the overall coordination of the programme and contributed to review and revision of the manuscript.

SJ, BO, JLP, CD coordinate the programme in India (SJ), Nigeria (BO) and Trinidad (JLP, CD) and contributed to review and revision of the manuscript.

CM led the design of the programme and the study methods, leads the programme, contributed to drafting and revising the manuscript, and provided guidance and supervision throughout the preparation of the manuscript.

Competing Interests Statement

RM has received payment for lectures from Janssen, Sunovian, Otsuka, Lundbeck, Angelini, and Rekordati.

Data Statement

The current article is a protocol for an ongoing study. When data are available, they will be shared upon reasonable request after submitting a synopsis of any proposed analyses to the study coordinator and receiving approval from the study PIs.

Acknowledgements

The authors wish to thank the research teams at the University of Ibadan, the Schizophrenia Research Foundation, and the University of the West Indies, for their ongoing work on this programme.

Figure legends

Figure 1: Structure of INTREPID II.

Figure 2: Summary of methodology.

For peer review only - http://bmjopen.bmj.com/site/about/guidelines.xhtml

For peer review only

For peer review only - http://bmjopen.bmj.com/site/about/guidelines.xhtml

Study	Baseline 720 cases; 720 controls	2 Year Follow-Up ~ 576 cases; ~ 576 controls
(1) Incidence and presentation	X	-
(2) Course and outcome	X	X
(3) Help-Seeking and Impact	X	X
(4) Physical Health	X	X

Figure 1. Structure of INTREPID II.

For peer review only

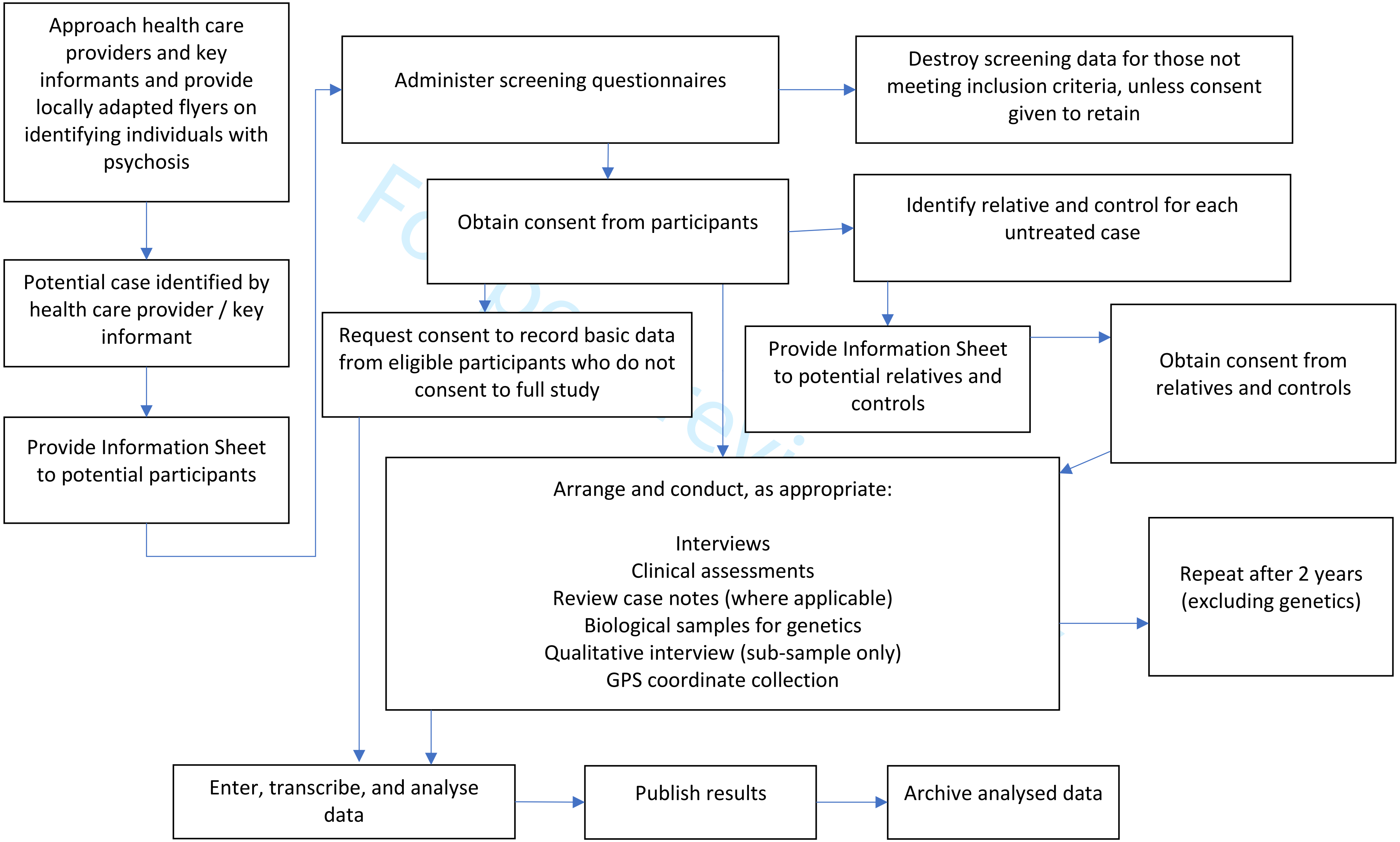

Figure 2. Summary of methodology.

For peer review only - http://bmjopen.bmj.com/site/about/guidelines.xhtml

Supplementary Materials

STROBE Statement—checklist of items that should be included in reports of observational studies

	Item No	Recommendation	Page No
Title and abstract	1	(a) Indicate the study's design with a commonly used term in the title or the abstract	2
		(b) Provide in the abstract an informative and balanced summary of what was done and what was found	2
Introduction			
Background/rationale	2	Explain the scientific background and rationale for the investigation being reported	4
Objectives	3	State specific objectives, including any prespecified hypotheses	4,5,6,7
Methods			
Study design	4	Present key elements of study design early in the paper	8
Setting	5	Describe the setting, locations, and relevant dates, including periods of recruitment, exposure, follow-up, and data collection	7-8
Participants	6	(a) Cohort study—Give the eligibility criteria, and the sources and methods of selection of participants. Describe methods of follow-up<br>Case-control study—Give the eligibility criteria, and the sources and methods of case ascertainment and control selection. Give the rationale for the choice of cases and controls<br>Cross-sectional study—Give the eligibility criteria, and the sources and methods of selection of participants	8-10, table 1
		(b) Cohort study—For matched studies, give matching criteria and number of exposed and unexposed<br>Case-control study—For matched studies, give matching criteria and the number of controls per case	8-9, table 1
Variables	7	Clearly define all outcomes, exposures, predictors, potential confounders, and effect modifiers. Give diagnostic criteria, if applicable	Table 2
Data sources/ measurement	8*	For each variable of interest, give sources of data and details of methods of assessment (measurement). Describe comparability of assessment methods if there is more than one group	10-11, Table 2
Bias	9	Describe any efforts to address potential sources of bias	10-11

For peer review only

Study size	10	Explain how the study size was arrived at	10
Quantitative variables	11	Explain how quantitative variables were handled in the analyses. If applicable, describe which groupings were chosen and why	N/A (programme protocol covering many research questions)
Statistical methods	12	(a) Describe all statistical methods, including those used to control for confounding	11-12
		(b) Describe any methods used to examine subgroups and interactions	11
		(c) Explain how missing data were addressed	11
		(d) Cohort study—If applicable, explain how loss to follow-up was addressed<br>Case-control study—If applicable, explain how matching of cases and controls was addressed<br>Cross-sectional study—If applicable, describe analytical methods taking account of sampling strategy	11
		(e) Describe any sensitivity analyses	11
Results			
Participants	13*	(a) Report numbers of individuals at each stage of study—eg numbers potentially eligible, examined for eligibility, confirmed eligible, included in the study, completing follow-up, and analysed	N/A (protocol)
		(b) Give reasons for non-participation at each stage	N/A (protocol)
		(c) Consider use of a flow diagram	N/A (protocol)
Descriptive data	14*	(a) Give characteristics of study participants (eg demographic, clinical, social) and information on exposures and potential confounders	N/A (protocol)
		(b) Indicate number of participants with missing data for each variable of interest	N/A (protocol)
		(c) *Cohort study*—Summarise follow-up time (eg, average and total amount)	N/A (protocol)
Outcome data	15*	*Cohort study*—Report numbers of outcome events or summary measures over time	N/A (protocol)
		Case-control study—Report numbers in each exposure category, or summary measures of exposure	N/A (protocol)

For peer review only

		Cross-sectional study—Report numbers of outcome events or summary measures	N/A (protocol)
Main results	16	(*a*) Give unadjusted estimates and, if applicable, confounder-adjusted estimates and their precision (eg, 95% confidence interval). Make clear which confounders were adjusted for and why they were included	N/A (protocol)
		(*b*) Report category boundaries when continuous variables were categorized	N/A (protocol)
		(*c*) If relevant, consider translating estimates of relative risk into absolute risk for a meaningful time period	N/A (protocol)
Other analyses	17	Report other analyses done—eg analyses of subgroups and interactions, and sensitivity analyses	N/A (protocol)
Discussion			
Key results	18	Summarise key results with reference to study objectives	N/A (protocol)
Limitations	19	Discuss limitations of the study, taking into account sources of potential bias or imprecision. Discuss both direction and magnitude of any potential bias	N/A (protocol)
Interpretation	20	Give a cautious overall interpretation of results considering objectives, limitations, multiplicity of analyses, results from similar studies, and other relevant evidence	N/A (protocol)
Generalisability	21	Discuss the generalisability (external validity) of the study results	N/A (protocol)
Other information			
Funding	22	Give the source of funding and the role of the funders for the present study and, if applicable, for the original study on which the present article is based	1

*Give information separately for cases and controls in case-control studies and, if applicable, for exposed and unexposed groups in cohort and cross-sectional studies.

Note: An Explanation and Elaboration article discusses each checklist item and gives methodological background and published examples of transparent reporting. The STROBE checklist is best used in conjunction with this article (freely available on the Web sites of PLoS Medicine at http://www.plosmedicine.org/, Annals of Internal Medicine at http://www.annals.org/, and Epidemiology at http://www.epidem.com/). Information on the STROBE Initiative is available at www.strobe-statement.org.

For peer review only

Appendix 1: Feasibility and Pilot Work

To design and test robust methods to investigate our hypotheses in diverse settings, we conducted extensive feasibility and pilot work in catchment areas in each of the three settings. This consisted of three components. First, we conducted a mapping exercise to identify and engage all professional and folk (traditional) providers and potential key informants within a defined catchment area (approximately half of the area to be covered by INTREPID II), to create a locally tailored case detection system through which to identify and recruit representative samples of cases of psychosis (35). Second, we conducted qualitative research to understand how psychoses are conceptualised locally, in order to facilitate case identification (5). This allowed us to develop locally relevant materials for service providers and key informants identified through our mapping exercise, based on an in-depth understanding of the terminology used to refer to people experiencing psychotic symptoms, the outward manifestations of psychosis that are frequently observed in this context, and patterns of help-seeking. Third, we implemented the methods to be used in INTREPID II for 6-7 months in these catchment areas to assess their feasibility (1-3). This included testing methods for identifying and recruiting age- and sex-matched controls (i.e. non-psychotic individuals) and for following both cases and controls over time.

The pilot project demonstrated that it was possible to identify and recruit both cases and controls through our local detection systems, and to collect extensive data from these participants using the proposed instruments In the process, it established the infrastructure necessary for the INTREPID II programme to conduct larger scale research using these methods in order to test our primary hypotheses. The findings from each of these stages have been described in detail elsewhere (1-3).

References

1. Morgan C, Hibben M, Esan O, John S, Patel V, Weiss HA, et al. Searching for psychosis: INTREPID (1): systems for detecting untreated and first-episode cases of psychosis in diverse settings. Social psychiatry and psychiatric epidemiology. 2015;50(6):879-93.
2. Morgan C, John S, Esan O, Hibben M, Patel V, Weiss H, et al. The incidence of psychoses in diverse settings, INTREPID (2): a feasibility study in India, Nigeria, and Trinidad. Psychological medicine. 2016;46(9):1923-33.
3. Cohen A, Padmavati R, Hibben M, Oyewusi S, John S, Esan O, et al. Concepts of madness in diverse settings: a qualitative study from the INTREPID project. BMC psychiatry. 2016;16(1):388.

For peer review only

Appendix 2: Ongoing and Planned Extensions

In addition to enabling us to investigate and test our primary research questions and hypotheses, INTREPID II establishes in each setting platforms and infrastructure for the conduct of other studies. Building on this, several extensions to INTREPID II are ongoing or planned. Here we highlight four.

(1) Data pooling for international comparisons

A particularly valuable aspect of INTREPID II is that our measures and methods are aligned with previous major research programmes on psychotic disorders in Europe to enable direct comparisons and data pooling to explore variation between countries and populations. For example, secondary hypotheses, regarding incidence rates, initial presentation, and risk factors will be tested by combining INTREPID II data with data from the UK, the Netherlands, France, Spain and Italy collected as part of the AESOP and EU-GEI studies (e.g., that, overall, incidence rates will be lower in India and Nigeria than in Trinidad and northern European countries).

(2) Genetics

Our current understanding of the genetics of psychotic disorders is limited by lack of diversity in the samples used. Most samples comprise individuals of European ancestry. Consequently, when applied to other populations, findings are less applicable. For example, polygenic risk scores (PRS), a measure of the total effects of multiple genes on risk (1), derived from large scale genome-wide association studies, explain far less of the variance in risk when applied in non-European samples (2). INTREPID II, then, provides an opportunity to generate samples from diverse populations that can contribute to global efforts to expand genetic studies to include people of African, Caribbean, and Asian ancestry.

Extending what we originally planned, participating cases and controls will now be invited to provide DNA samples at baseline via blood or saliva samples. Those who provide informed consent will have 10ml blood samples collected in EDTA tubes by a phlebotomy-trained researcher, or 2.5ml of saliva can be provided using Oragene saliva kits. Samples will be shipped to our partner organisations in the USA for DNA extraction and genome-wide association studies (GWAS), with extracted DNA samples then returned to the study settings. By pooling these data with larger datasets, our data will contribute to the generation of PRS that are applicable to our target populations, which we can then use to analyse genetic risk and gene-environment interactions within the INTREPID II cohort.

(3) Spatial Effects

The use of accessible technology to collect GPS coordinates for all participants, service providers and traditional healers provides an opportunity to explore spatial effects – in incidence, risk factors, outcomes, and help-seeking

– at the neighbourhood level. This will allow us to link individual data with ecological data in order to investigate risk and protective factors at the neighbourhood level, as well as facilitating geographical analyses of help-seeking behaviour.

(4) A Global Consortium

INTREPID II will act as a platform for the development of long-term international collaborations to address the lack of evidence on psychotic disorders in the Global South. INTREPID II provides a methodological template for epidemiological research on psychosis across diverse contexts, as exemplified by the establishment of a new research programme in South Africa that uses parallel methods, PSYMAP-ZN (led by Professor Bonginkosi Chiliza, University of KwaZulu-Natal, and Professor Jonathan Burns, University of Exeter). Building on these two programmes, we intend to establish a global consortium for population-based research involving INTREPID II and PSYMAP-ZN researchers and leading psychosis researchers from strategically-chosen settings in more diverse settings across the world – with a particular emphasis on areas where evidence is currently lacking – to extend this research agenda across geographic and disciplinary boundaries. The consortium will involve capacity building, data pooling, knowledge sharing platforms, the development of shared instruments and innovative methods, and will be underpinned by partnerships with service users and carers.

References

1. Consortium IS. Common polygenic variation contributes to risk of schizophrenia and bipolar disorder. Nature. 2009;460(7256):748.
2. Vilhjálmsson BJ, Yang J, Finucane HK, Gusev A, Lindström S, Ripke S, et al. Modeling linkage disequilibrium increases accuracy of polygenic risk scores. The American Journal of Human Genetics. 2015;97(4):576-92.

For peer review only
