# Supplementary material for: INTREPID II: protocol for a multistudy programme of research on untreated psychosis in India, Nigeria and Trinidad
Source: BMJ Open. 2020 Jun 21;10(6):e039004. doi: 10.1136/bmjopen-2020-039004 (PMC7311008; doi:10.1136/bmjopen-2020-039004)
Supplement: Reviewer comments [file bmjopen-2020-039004.reviewer_comments.pdf]

PEER REVIEW HISTORY

BMJ Open publishes all reviews undertaken for accepted manuscripts. Reviewers are asked to complete a checklist review form (**http://bmjopen.bmj.com/site/about/resources/checklist.pdf**) and are provided with free text boxes to elaborate on their assessment. These free text comments are reproduced below.

ARTICLE DETAILS

**TITLE (PROVISIONAL)**	INTREPID II: Protocol for a multi-study programme of research on untreated psychosis in India, Nigeria, and Trinidad
**AUTHORS**	Roberts, Tessa; Gureje, Oye; Thara, Rangaswamy; Hutchinson, Gerard; Cohen, Alex; Weiss, Helen; John, Sujit; Lee Pow, Joni; Donald, Casswina; Olley, Bola; Miguel Esponda, Georgina; Murray, Robin; Morgan, Craig

This article was not externally reviewed at BMJ Open. Protocols that have been independently assessed prior to submission to BMJ Open are usually fast-tracked to publication on the grounds that further substantial changes will not be possible. This independent assessment will usually be external, independent review for both a substantial grant award from a non-commercial or government funder and ethics approval.
